# A novel *ND1* mitochondrial DNA mutation is maternally inherited in growth hormone transgenesis in amago salmon (*Oncorhynchus masou ishikawae*)

**DOI:** 10.1038/s41598-022-10521-4

**Published:** 2022-04-25

**Authors:** Tomohiko Sato, Naoko Goto-Inoue, Masaya Kimishima, Jike Toyoharu, Ryuhei Minei, Atsushi Ogura, Hiroyuki Nagoya, Tsukasa Mori

**Affiliations:** 1 Department of Marine Science and Resources, Nihon University College of Bioresource Sciences, Kameino 1866, Fujisawa, 252-0880 Japan; 2 Research Institute of Medical Research Support Center Electron Microscope Laboratory, School of Medicine, Nihon University, Tokyo, 173-8610 Japan; 3 Department of Computer Bioscience, Nagahama Institute of Bio-Science and Technology, Nagahama, 526-0829 Japan; 4 National Research Institute of Aquaculture, Fisheries Research and Education Agency, Minamiise, 516-0193 Japan

**Keywords:** Biochemistry, Genetics, Physiology

## Abstract

Growth hormone (GH) transgenesis can be used to manipulate the growth performance of fish and mammals. In this study, homozygous and hemizygous GH-transgenic amago salmon (*Oncorhynchus masou ishikawae*) derived from a single female exhibited hypoglycemia. Proteomic and signal network analyses using iTRAQ indicated a decreased NAD^+^/NADH ratio in transgenic fish, indicative of reduced mitochondrial *ND1* function and ROS levels. Mitochondrial DNA sequencing revealed that approximately 28% of the deletion mutations in the GH homozygous- and hemizygous-female-derived mitochondrial DNA occurred in *ND1*. These fish also displayed decreased ROS levels. Our results indicate that GH transgenesis in amago salmon may induce specific deletion mutations that are maternally inherited over generations and alter energy production.

## Introduction

Gene manipulation technology is commonly used in various fields such as agriculture, aquaculture, horticulture, and medical science. Growth hormone (GH) has been used for growth enhancement in animals, as it is involved in the metabolism of carbohydrates, lipids, nitrogen, and minerals^1^, as well as in cell differentiation^2^, maintenance of the immune system^3^, cardiac function^4^, suppression of emotions, stress responses, and behavior^5^.

GH transgene technology has been widely employed at the research in the aquaculture of carp, tilapia, pond loach, red sea bream, zebrafish, coho salmon, rainbow trout, and Atlantic salmon^6^. In particular, GH-transgenic salmonids have been reported to exhibit growth rates that are 6- to 40-fold faster than those of control salmonids ^7,8^. In 2015, the Food and Drug Administration approved a growth-enhanced transgenic line of salmon for human consumption, leading to the commercialization of these fish (https://www.fda.gov/media/93801/download).

GH-transgenic Atlantic salmon exhibit distinctive phenotypic characteristics compared with those of wild-type fish, including increased gill surface area^9^, development of intestinal villi^10^, increased food conversion efficiency^6,7^, and utilization of lipids in ingested food^11^. Side effects of GH overexpression can include acromegaly^12^, cardiac hypertrophy^13^, and decreased fertility^6^. However, adverse effects vary among lines and species and are not observed consistently. In a previous study, we observed shrinkage in the pituitary gland owing to the negative feedback of GH in GH-transgenic salmon^14^. The pituitary gland produces GH in addition to other hormones that enable animals to adapt to diverse environmental conditions.

We successfully established a GH-transgenic amago salmon (*Oncorhynchus masou ishikawae*) line by injecting eggs from a single female GH-transgenic fish with a salmon GH construct, *OnMTGH1*^7^ (*Oncorhynchus masou* GH1 gene fused to metallothionein-B), and subsequently demonstrated the downregulation of Δ6-desaturase, apolipoprotein, pentraxin, and serum lysozyme activity in the transgenics^15^. These transgenic fish exhibited morphological changes in the liver tissues^16^, significantly elevated concentrations of plasma GH1 and insulin-like growth factor 1, and decreased total cholesterol and triglyceride levels^17^ compared to wild-type fish. Moreover, homozygous (ectopic GH, Tg derived from both female and male, ♀/♂, Tg/Tg) and hemizygous GH-transgenic (ectopic GH derived from the female fish, Tg/+) fish exhibit decreased serum glucose levels, increased *GRP78* (glucose starvation gene) expression, and increased ketone body (3-hydroxybutyric acid) levels^18^, as well as decreased levels of saturated fatty acids (14:0) and monounsaturated fatty acids (16:1n-7, 18:1n-9) in the liver and muscle tissues^19^.

It is unclear whether the altered physiological phenotypes observed in GH-transgenic fish stem solely from genomic variations or altered mitochondrial functions. It is generally believed that transgenesis influences only the expression of genes in the nuclear genome of the host. However, there is no evidence of abnormalities in GH-transgenic amago salmon derived solely from genomic differences, and research concerning this aspect may be crucial for the safety assessment of transgenesis. Therefore, we first investigated the physiological differences between hemizygous fish whose ectopic DNA originated from males and females. We found differences in the serum glucose concentration between (Tg/+) and (+/Tg) (ectopic GH from the male) fish, with the pattern being reproduced in a subsequent generation. These results suggest a role for mitochondrial DNA (mtDNA) in the physiology of GH-transgenic amago salmon. Therefore, we examined whether mitochondrial dysfunction contributes to these phenotypes in GH-transgenic fish.

## Results

### Generation of GH-transgenic fish

We injected On*MTGH1*^7^, a salmon GH construct, into approximately 5000 eggs and obtained 13 mosaic fish and 1 female fish with the inherited, ectopically integrated On*MTGH1* in the germ cells. The GH-transgenic amago salmon line was generated using this single female fish (Tg/+), and the female GH-transgenic fish (Tg/+) was fertilized by wild-type males (+/+) to generate F1 GH-transgenic fish (Tg/+) and (+/+). Thereafter, the F1 fish were selected by PCR using On*MTGH1* sequencing primers (Eurofins Genomics Inc, Tokyo)^18^. The selected transgenic F1 fish (Tg/+) were fertilized with each other to generate (Tg/Tg). The copy number of GH transgenes in (Tg/Tg), hemizygous fish (Tg/+), and (+/Tg) fish was determined using real-time PCR^18^, and 14 copies of the gene were introduced into (Tg/+) fish. The selected (Tg/Tg) fish were fertilized with wild eggs and wild sperm to produce (Tg/+) and (+/Tg), respectively. If the sperm and eggs of the homozygous fish (Tg/Tg) were fertilized by wild-type fish (+/+), and 100% of the hemizygous recombinants were confirmed by real-time PCR, the male and female fish were considered perfect homozygous fish (Tg/Tg). However, if the recombinant and non-recombinant fish appeared in a 1:1 ratio, they were considered hemizygous.

**Figure 1:**
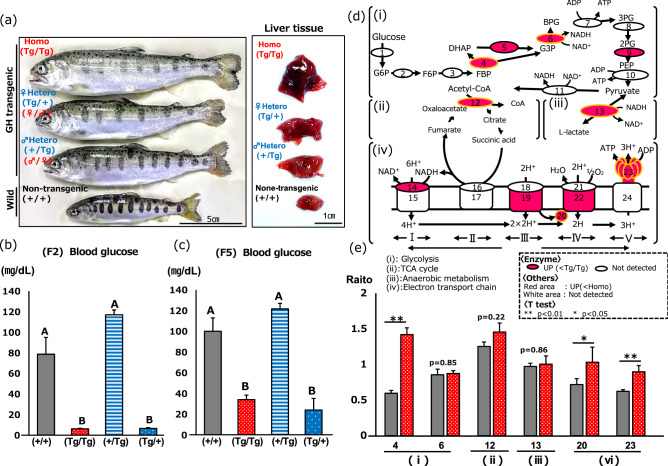
Generation of the GH-transgenic fish and iTRAQ proteomics. (**a**) GH-transgenic and non-transgenic fish used for the experiments. (Tg/+) indicates that the fish is the result of fertilization of (Tg/Tg) eggs and (+/+) sperm. (+/Tg) indicates that the fish is the result of fertilization of (+/+) eggs and (Tg/Tg) sperm. (Tg/Tg) and (+/+) fish were used for iTRAQ and mtDNA analysis. (**b** and **c**) Measurement of blood sugar concentration in different generations (F2 and F5). Different letters indicate a significant difference (*p* < 0.05; one-way ANOVA followed by Dunnett T3 test (F2) and Bonferroni (F5). F2, *n* = 6; (+/+), *n* = 10; (Tg/Tg), *n* = 10; (+/Tg), *n* = 10; (Tg/+). F5, *n* = 4; (+/+), *n* = 8; (Tg/Tg), *n* = 5; (+/Tg), *n* = 5 (Tg/+). (**d**) Mapping of detected proteins by iTRAQ into glycolysis (i), TCA (ii), anaerobic metabolism (iii), and the electron transport chain (iv). Red circles and red coloring denote the upregulation of proteins relative to non-transgenic siblings. Blank circles and white color denote proteins that were not detected by iTRAQ. Orange circles represent proteins confirmed by western blotting. (**e**) Signal intensity in western blots. (See “Supplementary Information” and “Supplementary figures” for the mapped enzymes indicated in **e**). Red denotes homozygous (Tg/Tg) fish, and gray denotes non-transgenic (+/+) fish. *p < 0.05; **p < 0.01. Vertical bars represent SEM.

Homozygous GH-transgenic salmon were denoted as (♀/♂; Tg/Tg); hemizygous GH-transgenic salmon where the ectopic GH was derived from the female were denoted as (♀/♂; Tg/+), and transgenic fish where the ectopic GH was derived from the male were denoted as (♀/♂; +/Tg). Non-GH-transgenic siblings were denoted as (+/+). There were distinct size differences among the homozygous and hemizygous transgenic juvenile fish (Fig. 1a). Furthermore, abnormal morphogenesis was observed in liver tissues of GH-transgenic fish over generations, exhibiting irregular and complex shapes in (Tg/Tg), (Tg/+), and (+/Tg) fish. Specifically, the surface of liver tissues derived from (Tg/Tg) fish was uneven, with abnormal fissures and evidence of vasodilatation. In contrast, non-transgenic liver tissues were elliptical and had a smooth surface. Blood glucose concentration was unchanged (*p* = 0.05) between (+/+) and (+/Tg) fish. However, blood glucose levels in (Tg/Tg) and (Tg/+) fish were significantly lower than those in (+/+) fish through the F2 and F5 generations (Fig. 1b,c).

### Isobaric tags for relative and absolute quantitation (iTRAQ)-based proteomics

Subsequently, we employed proteomics to identify metabolic pathways associated with glycolysis and ATP production. Liver tissues from (Tg/Tg) and (+/+) fish were used for this analysis as (Tg/Tg) fish displayed low serum glucose levels and morphological abnormalities in the liver. Of the 292 proteins identified, 270 exhibited a 1.5-fold or greater increase in expression in (Tg/Tg) fish compared with that in (+/+) fish (Supplementary Table 1). These upregulated proteins were involved in glycolysis, the tricarboxylic acid (TCA) cycle, anaerobic metabolism, and the electron transport chain (Fig. 1d, Table 1). Compared with (+/+) fish, (Tg/Tg) fish showed upregulated (1.4- to 6.7-fold) expression of four out of ten glycolytic enzymes, including alpha-enolase and fructose-bisphosphate aldolase A (Fig. 1e; i, Table 1); a twofold increase in the expression of citrate synthase, which converts acetyl-CoA to citrate in the TCA cycle (Fig. 1d; ii); a 1.8-fold increase in l-lactate dehydrogenase levels (Fig. 1d; iii); and a 1.6- to 2.8-fold increase in the expression of complexes I, II, IV, and V of the mitochondrial electron transport chain (Fig. 1d; iv and Supplementary Table 1). We performed western blotting to confirm these data. Fructose-bisphosphate aldolase A, cytochrome c, and ATP synthase (ATP5a) levels in (Tg/Tg) fish were significantly higher (*p* < 0.01, *p* < 0.05, and *p* < 0.01, respectively) than those in (+/+) fish (Fig. 1e, “Supplementary Information”).

**Table 1:** Glycolysis-, aerobic-metabolism-, anaerobic-metabolism-, and electron-transport-chain-related proteins.

Number	Description	Score	Fold change (homo/control)
	**(a) Glycolysis**		
4	Fructose-bisphosphate aldolase A	162.3	6.7
4	Fructose-bisphosphate aldolase B	1461.4	1.7
5	Triosephosphate isomerase	8338.1	1.6
6	Glyceraldehyde-3-phosphate dehydrogenase	502.1	1.9
9	Alpha-enolase	8182.4	1.4
9	Beta-enolase	5600.8	1.5
	**(b) TCA cycle**		
12	Citrate synthase, mitochondrial	263.8	2
	**(c) Anaerobic metabolism**		
13	l-Lactate dehydrogenase B chain	1853	1.8
	**(d) Electron transport system related proteins**		
	Complex I		
14	NADH-ubiquinone oxidoreductase chain 1	31.9	2.2
14	NADH-ubiquinone oxidoreductase chain 2	88.14	2.2
14	NADH-ubiquinone oxidoreductase chain 5	41.43	2.8
	Complex III		
17	Cytochrome b	0	1.9
	Complex IV		
20	Cytochrome c	1542.2	2.1
22	Cytochrome c iso-1/iso-2	479.6	1.6
22	Cytochrome c oxidase subunit submit 1	253.2	1.9
22	Cytochrome c oxidase subunit 2	453.2	2.5
22	Cytochrome c oxidase subunit 4 isoform 2	131.2	2
22	Cytochrome c oxidase subunit 5A-2	264.1	1.6
22	Cytochrome c oxidase subunit 6A	63.2	2
22	Cytochrome c oxidase submit 7c	49.1	1.6
	Complex V		
23	ATP synthase protein 8	73.3	2.2
23	ATP synthase subunit β	13,627.3	1.8

### Ingenuity pathway analysis (IPA) of iTRAQ data

**Figure 2:**
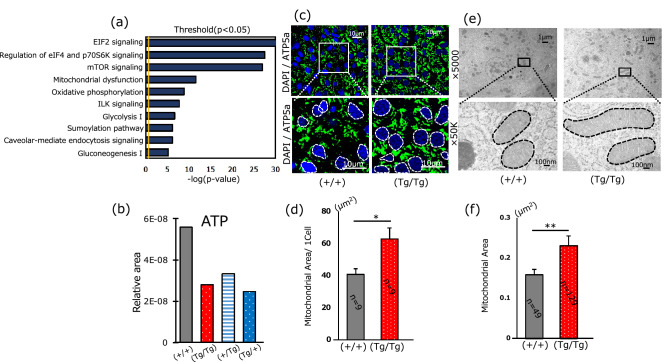
Signal transduction analysis using IPA and transmission electron micrography of mitochondria. (**a**) Top 10 signal transduction networks in the liver tissue from (Tg/Tg) fish based on iTRAQ data. (**b**) The concentration of ATP using five livers from GH homozygous and hemizygous fish and non-transgenic siblings. Bars represent the mean values for five samples. Five livers were pooled as one sample. Relative area = Peak area of the target sample/[Area value of internal standard × Sample weight (mg)]. (**c**) Mitochondrial observation by immunofluorescence against ATP5a (Green) and DAPI (Blue) [(+/+), (Tg/Tg): each *n* = 3). (**d**) Signal intensity corresponding to ATP5a. Three fields from one slide were randomly selected, and fluorescence was quantified. The area was divided by the number of nucleoli. Statistical significance was obtained using Student’s *t*-test. (**e**) Transmission electron micrograph of mitochondria; the black square (× 5000) was magnified 50,000 times. [(+/+), (Tg/Tg): each *n* = 3).The mitochondrial outline is defined by dotted lines, and (**f**) the area was measured using Image J. Statistical significance was obtained using Student’s *t*-test.

Signal transduction networks were analyzed to identify the signaling cascades affected by GH transgenesis. The amino acid sequences of the proteins identified in this study were aligned with those of the proteins available in the UniProt database (14 putative fish-specific proteins did not match the corresponding IDs in the database), and IPA revealed that mitochondrial dysfunction was among the top five altered metabolic pathways (Fig. 2a and Supplementary Table 2). Furthermore, disease and disorders, physiological system development and function, and molecular and cellular function were included in the top five altered pathways (Table 2). In the disease and disorders category, proteins related to cancer, organismal injury, and neurological diseases were expressed in GH-homozygous fish. In addition, proteins involved in cellular enlargement, such as cardiovascular components and organ development in the physiological development category, were also expressed.

**Table 2:** Summary of IPA prediction for diseases and disorders, physiological system development and function, molecular and cellular function, and top tox using iTRAQ data.

Disease and disorders			Molecular and cellular function		
Name	P-value range	Molecules	Name	P-value range	Molecules
Cancer	7.92E − 04 to 4.57E − 28	95	Cell death and survival	7.06E − 04 to 4.57E − 28	67
Organismal injury and abnormalities	7.92E − 04 to 4.57E − 28	97	Protein synthesis	3.23E − 04 to 1.26E − 18	32
Tumor morphology	2.39E − 21 to 4.57E − 28	29	Gene expression	7.32E − 04 to 3.84E − 13	41
Neurological disease	7.41E − 04 to 1.22E − 11	47	Cell cycle	7.97E − 04 to 9.34E − 10	31
Psychological disorders	5.30E − 04 to 1.22E − 11	40	RNA post-transcriptional modification	4.76E − 04 to 1.38E − 07	11

Physiological system development and function			Top TOX lists		Overlap
Cardiovascular system development	6.22E − 04 to 6.98E − 07	20	Aryl hydrocarbon receptor signaling	7.14E − 08	5.7% 9/159
Organ development	7.73E − 04 to 1.27E − 06	16	Renal necrosis/cell death	4.55E − 05	2.1% 11/52
Organ morphology	5.63E − 04 to 1.27E − 06	17	Cardiac hypertrophy	6.39E − 05	2.2% 10/45
Skeletal and muscular system development	7.74E − 04 to 1.27E − 06	14	Cytochrome P450 panel	8.21E − 05	16.7% 3/18
Endocrine system development	4.68E − 04 to 3.66E − 06	7	Mitochondrial dysfunction	2.02E − 04	3.4% 6/176

### Determination of ATP concentration and analysis of mitochondrial morphology by transmission electron microscopy (TEM) of liver tissues

**Figure 3:**
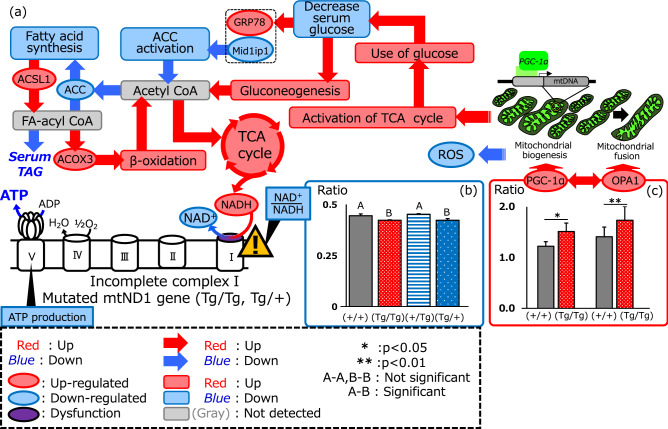
Physiological conditions of GH homozygous fish. (**a**) Summary of physiological conditions in the (Tg/Tg) and (Tg/+) fish. Differential expression of the glucose starvation gene (*GRP78*), MID1 interacting protein 1 (*MID1IP1*), A long-chain-fatty-acid-CoA ligase 1 (*ACSL1*), acetyl-CoA carboxylase (*ACC*), and acyl-CoA oxidase 3 (*ACOX3*) in (Tg/Tg) fish was obtained from Sugiyama et al.^18^. (**b**) The NAD^+^/NADH ratio was measured using LC–MS (*n* = 3: different fish), which was significantly decreased [*p* < 0.05; ANOVA followed by PLS. A–B indicate significant differences in (Tg/Tg) and (Tg/+) fish vs. control and (+/Tg) fish]. (**c**) Peroxisome proliferator-activated receptor γ coactivator-1 (*PGC-1α*) and mitochondrial dynamin-like GTPase (*OPA1*) levels were quantified by western blotting (See “Supplementary Information” and “Supplementary figures”). The intensities of the four *PGC-1α* and *OPA1* signals were divided by the β-actin signal; the vertical bars represent standard errors. Statistical significance was obtained by Student’s *t*-test. *p < 0.05; **p < 0.01.

The ATP concentration of (Tg/Tg) and (Tg/+) fish were reduced to less than half of that of (+/+) fish. In this experiment, we were unable to identify significant differences between individuals, as samples from five fish of the same genotype were pooled. iTRAQ data revealed a 1.9-fold increase in optic atrophy 1 (OPA1; a dynamin-like 120-kDa protein, mitochondrial) levels in (Tg/Tg) fish compared with that in (+/+) fish. While enhanced ATP synthesis was predicted based on the OPA1 levels, our data contradicted this (Fig. 2b). We determined the number and morphology of mitochondria using fluorescence-labeled anti-ATP5a antibodies (Fig. 2c) and observed a significant (*p* < 0.05) increase in the number of mitochondria per cell in (Tg/Tg) fish (Fig. 2d). We then used TEM to investigate mitochondrial morphology in transgenic fish (Fig. 2e). A significant difference (*p* < 0.01) in mitochondrial area and shape was observed between (Tg/Tg) and (+/+) fish (Fig. 2f). In Fig. 3a, the overall physiological conditions and phenotypic predictions for the GH-transgenic fish are summarized based on previously published data^18^. As shown in Fig. 3a, NADH is produced from NAD^+^ via the TCA cycle, and NADH is converted to NAD^+^ by complex 1 in mitochondria. Therefore, complex 1 plays a homeostatic role with respect to the NAD^+^/NADH ratio. The NAD^+^/NADH ratio in (Tg/Tg) and (Tg/+) fish was significantly lower than that in (+/+) and (+/Tg) fish (*p* < 0.05) (Fig. 3b). Furthermore, relationships between peroxisome proliferator-activated receptor γ coactivator-1 (PGC-1α), OPA1, and NAD^+^ were predicted by IPA (Fig. 3a). Low serum glucose levels resulted in enhanced gluconeogenesis and TCA cycle to compensate for low ATP concentrations; However, dysfunction of the ND1 (NADH-ubiquinone oxidoreductase chain 1) site results in decreased conversion of NADH to NAD^+^ and lower NAD^+^/NADH concentrations. Consequently, ATP production is reduced (Fig. 2b) and the TCA cycle is further activated to produce ATP. Western blotting revealed that PGC-1α expression was significantly higher (*p* < 0.05) in (Tg/Tg) fish than that in control fish (Fig. 3c), as predicted in Fig. 3a. Western blotting for OPA1 revealed five major protein signals (“Supplementary Information”), and the total signal intensity was significantly higher (*p* < 0.01) in (Tg/Tg) fish than that in (+/+) fish (Fig. 3c and “Supplementary Information”).

### Analysis of mtDNA and RNA expression

As a decreased NAD^+^/NADH ratio reflects dysfunctional *ND1* in the mitochondria, we analyzed the mtDNA sequences of homozygous and hemizygous transgenic fish. mtDNA sequencing and alignment were performed based on the genome sequence of *O. masou ishikawae* (NC_008746). We obtained 12,114,092 and 15,034,882 reads by 150 bp paired-end runs using (+/+) and (Tg/Tg) fish, respectively. Therefore, the coverage of the sequences against the mitochondria (16,652 bp) was 218,246- and 270,866-times, respectively.

**Figure 4:**
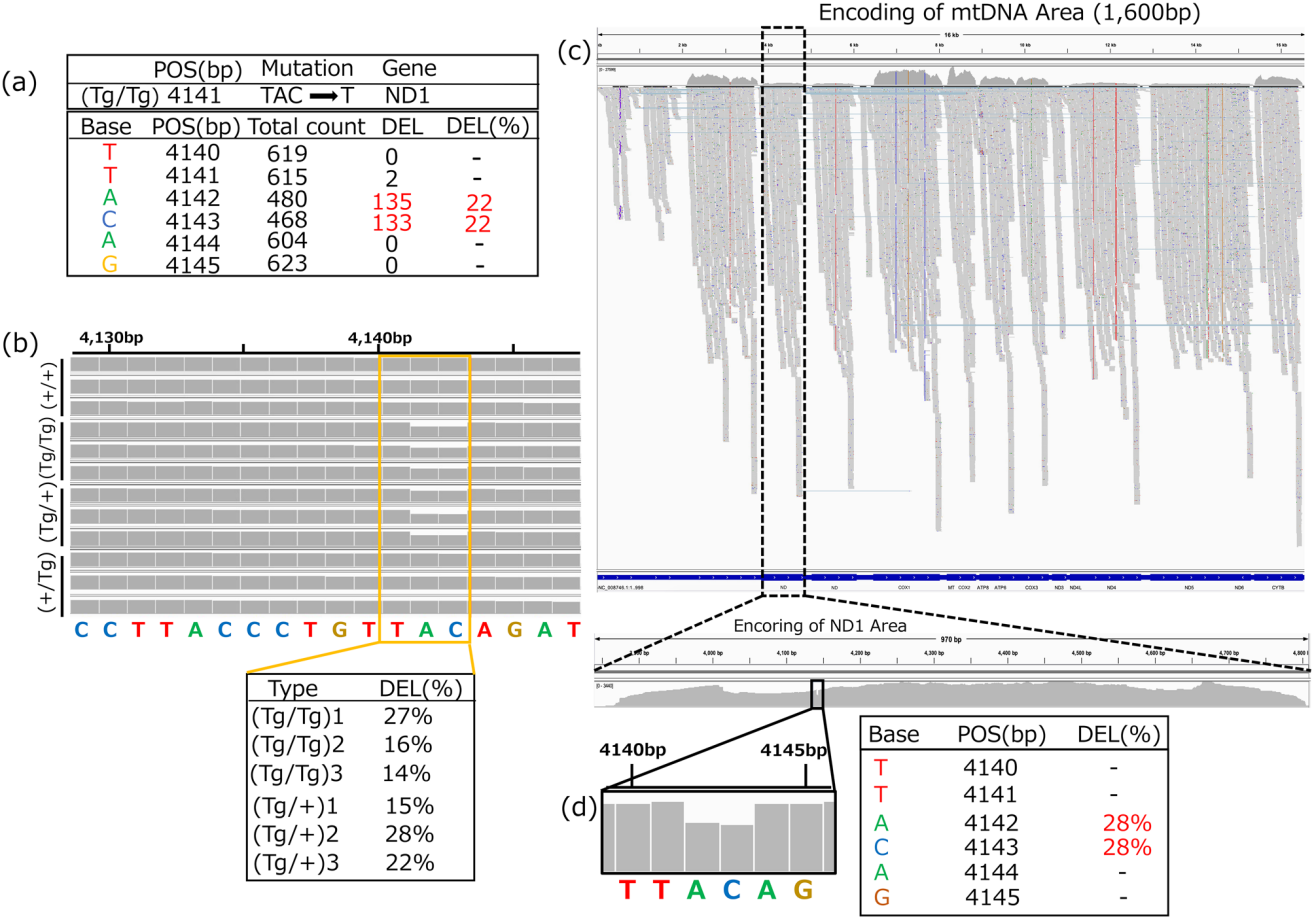
Analysis of mtDNA and mtRNA expression. (**a**) Mutation sites in mtDNA of (Tg/Tg) fish. “Total count” represents the total read count of no-deletion after eliminating duplication, and “DEL” denotes the read count containing the deletion mutation on the site. DEL % indicates that A and C were deleted at a rate of 22% [135/(480 + 135) × 100] and [133/(468 + 133) × 100] in the total mtDNA. (**b**) *ND1* site variant analysis using mtDNA. Whole *ND1* site sequencing was performed using three different fish. Type indicates fish, and DEL % denotes the average deletion mutation rate of A and C. (**c**) Validation of single nucleotide polymorphisms using RNA-seq. (**d**) The ratio of the deletion mutation in the ND1 mRNA of (Tg/Tg). Two fish from the (+/+) and (Tg/Tg) fish groups were used for whole mtDNA sequence, and three fish were used for the ND1 site sequence. One (Tg/Tg) fish was used for RNA seq for the ND1 site. (**c**, **d**) were created using Integrative Genomics Viewer (IGV: https://software.broadinstitute.org/software/igv/home) version 2.4.1.

Mutation analysis using the Genome Analysis Toolkit [GATK, a tool for identifying single nucleotide polymorphisms (SNPs) and indels] revealed four types of mutations. The “GC to G” and “C to T” mutations were detected in non-transgenic fish (+/+), whereas “C to CT” and “TAC to T” were detected in (Tg/Tg) fish. However, we focused our analysis on (Tg/Tg) fish as they exhibited the most significant morphological and metabolic abnormalities. The corresponding genes harboring these mutations were investigated, and “TAC to T” was found in *ND1* (Fig. 4a). Next, we performed a detailed investigation of the 4141 bp mtDNA sequence and observed that a deletion of “A” at position 4142 occurred in 44,346 (deletion count) of the 292,718 (total count) sequences, while “C” deletion at position 4143 occurred in 44,329 (deletion count) of the 293,437 (total count) sequences. Overlapping sequences were eliminated from these data, and deletions of “A” and “C” were present in 135 and 133 of the total count of 480 and 468 sequences (no-deletion base), respectively (Fig. 4a). These data indicate that 22% [134/(474+134) × 100] of the mtDNA harbored deletion mutations. We identified GH-transgenic fish harboring this deletion mutation by analyzing each of the three fish samples using Integrative Genomics Viewer (IGV) mapping (Fig. 4b). IGV mapping identified the deletion mutation in the 4142–4143 bp region in only the (Tg/Tg) and (Tg/+) fish (Fig. 4b). These mutation rates (DEL%) were similar in (Tg/Tg) and (Tg/+) fish (Fig. 4b).

We further investigated the effect of the deletion mutations at the *ND1* locus on RNA transcripts using RNA-seq (Fig. 4c) with RNA extracted from (Tg/Tg) and (+/+) fish. The RNA was mapped on the encoding mtDNA, and the *ND1* site was magnified (Fig. 4d). RNA was expressed up to the poly(A) site, yet the *ND1* site carried a deletion in the 4142–4143 bp region in all (Tg/Tg) fish. The mean total and deletion counts at the *ND1* site were 1556.5 and 609.5, respectively. Therefore, the proportion of deletions in *ND1* was 28%. These data suggest that mutated *ND1* in the mtDNA yields mutated *ND1* RNA in (Tg/Tg) and (Tg/+) fish.

### Analysis of mutated mitochondrial ND1 protein

**Figure 5:**
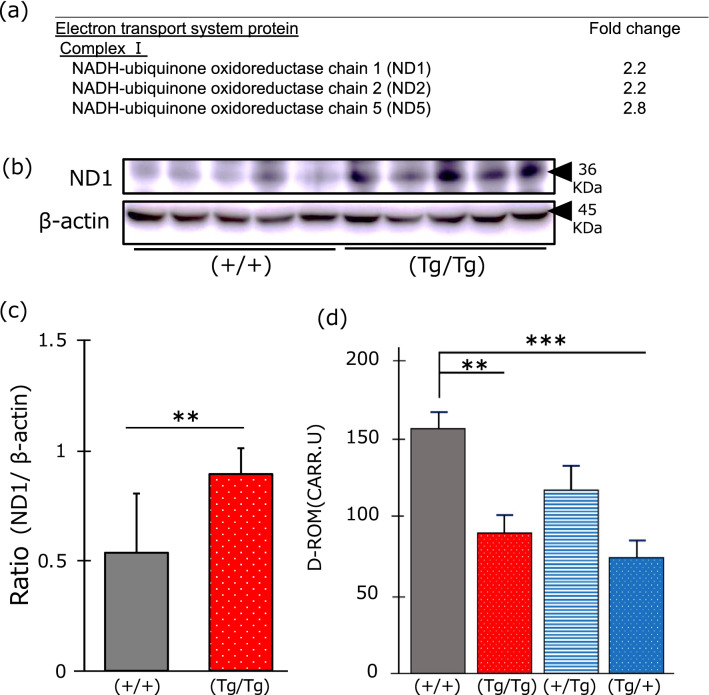
Analysis of mutated *ND1* in the mitochondria. (**a**) iTRAQ data of the electron transport system. (**b**) Western blotting for wild-type *ND1* and β-actin in (+/+) and (Tg/Tg) fish. This figure was created using Ingenuity Pathway Analysis (IPA) version 2.1, QIAGEN. (**c**) Signal intensities of ND1 proteins measured by ImageJ. Red denotes (Tg/Tg), and gray denotes (+/+). The *ND1* signal was divided by that of β-actin. (**d**) Concentration of ROS in (+/+), (Tg/Tg), (+/Tg), and (Tg/+) fish determined by d-ROM analysis. Vertical bars represent standard errors. **p < 0.01 and ***p < 0.001; Student’s *t*-test in (**c**) and one-way ANOVA followed by Bonferroni in (**d**). (See “Supplementary Information” about analyzed data of statistical analysis for the d-ROM test).

Mutated *ND1* RNA was expressed in (Tg/Tg) and (Tg/+) fish; therefore, we investigated whether the RNA was translated into protein. The molecular weight of the deduced *ND1* protein was 35.3 kDa, whereas that of the deduced mutated (TAC to T) *ND1* protein was 11.1 kDa. As several stop codons were predicted in the mutated *ND1*, we used an anti-*ND1* antibody that recognizes the N-terminal of *ND1*. In the iTRAQ experiment, expression of the *ND1* protein in (Tg/Tg) fish was 2.2-fold higher than that in (+/+) fish (Fig. 5a). Western blots revealed significantly higher (2.2-fold increase) expression of wild-type *ND1* (*p* < 0.01) in the (Tg/Tg) fish than that in the controls (+/+) (Fig. 5b,c). Immunoprecipitation using an anti-*ND1* antibody was performed to detect mutated *ND1*, after which all proteins were excised from the SDS–polyacrylamide gel and analyzed by MS/MS (Tandem mass spectrometer). However, no peptide corresponding to the mutant *ND1* peptide was detected after immunoprecipitation and re-analysis of the iTRAQ data.

### Measurement of oxidative stress using the diacron-reactive oxygen metabolite (d-ROM) test

The total amount of reactive oxygen species (ROS)-oxidized compounds was measured using the d-ROM test (Fig. 5d). Total ROS levels originating from (Tg/Tg) and (Tg/+) fish were significantly lower than those originating from (+/+) and (+/Tg) fish. This result was in line with the IPA prediction, based on the upregulation of PGC-1α (Fig. 3c). There were no significant differences in ROS levels between (+/+) and (+/Tg) fish (Fig. 5d).

### Deletion mutation sites in mtDNA and antioxidant defense systems

**Figure 6:**
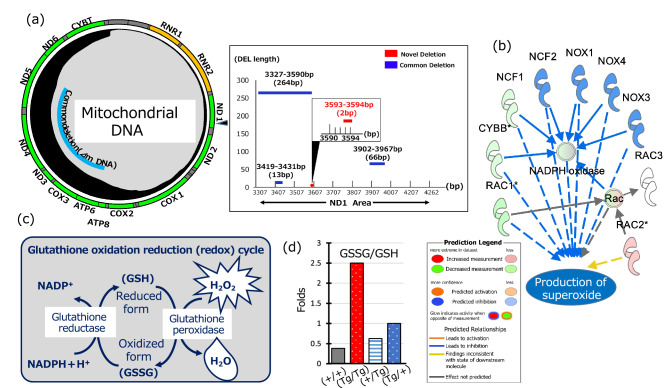
mtDNA mutational analysis and ROS levels. (**a**) *ND1* mutation site in (Tg/Tg) fish corresponding to human mtDNA was analyzed using the mtDNA breakpoints database (Mito Break; mitobreak.portugene.com). The black line in the circle (mtDNA) shows a historical record of mtDNA mutations. The black rectangle indicates the novel mutation. (**b**) Re-analysis of gene expression in (Tg/Tg) and (+/+) fish using previously reported data^18^ using IPA. This figure was created using Ingenuity Pathway Analysis (IPA) Content version: 57662101, QIAGEN (https://qiagen.secure.force.com/KnowledgeBase/KnowledgeIPAPage). Relationship between NADPH oxidase gene and superoxide production as predicted by IPA. *NCF1* (0.99-fold downregulation), *CYBB* (0.77-fold downregulation), *RAC1* (0.58-fold downregulation), and *RAC2* (1.46-fold upregulation) are components of NADPH oxidase. The asterisk indicates multiple IDs in the data set, and the data are displayed as the mean. Double circles indicate complexes of multiple genes. (**c**) Summary of the glutathione oxidation–reduction cycle. (**d**) Ratio of GSSG/GSH in (+/+), (Tg/Tg), (+/Tg), and (Tg/+) fish using capillary electrophoresis–mass spectrometry. The concentrations of GSSG and GSH in these fish are listed in “Supplementary Information”.

We investigated whether the deletion mutation sites identified in the mtDNA of (Tg/Tg) and (Tg/+) fish had previously been reported. We first performed a Basic Local Alignment Search Tool (BLAST) search of the (Tg/Tg) deletion mutation sites with those of humans as a reference. The amino acid residue “Thr” corresponded to Val in human *ND1* at the 96th position. Subsequently, we searched the mtDNA nucleotide site corresponding to the 96th amino acid residue using the human mitochondrial genome SNP database^20^. The deletion mutation (4142–4143 bp) observed in GH-transgenic amago salmon corresponded with that of 3593–3594 bp in human *ND1* (Fig. 6a). Thus, the deletion mutation detected in our transgenic fish was confirmed to be a novel mtDNA mutation based on the data hosted on the mtDNA breakpoints database.

Re-analysis of gene expression based on previous data^18^ revealed that the expression of components of NADPH oxidase, including neutrophil cytosolic factor 1 (*NCF1*), cytochrome b-245 beta chain (*CYBB*), Rac family small GTPase 1 (*RAC1*), and *RAC2*, were downregulated in (Tg/Tg) fish compared with that in (+/+) fish (Fig. 6b). These data also support the predicted decrease in superoxide levels in (Tg/Tg) and (Tg/+) fish (Fig. 3a), which was later confirmed (Fig. 5d). In multiple comparisons using RNASeq, data are generally analyzed using statistical selection with 10-fold/0.5-fold up/down control or FDR as a biologically significant cutoff. However, in the present study, we weakened the statistical cutoff and conducted IPA using as much data as possible to obtain an overview of the physiological changes in GH transgenic fish.

In addition, statistical analysis of the physiological phenomena and protein changes deduced from these genetic changes were used to investigate the process inside the body of GH transgenic amago salmon.

Superoxide is converted into H_2_O_2_ by superoxide dismutase, and H_2_O_2_ is further reduced to H_2_O during the conversion of glutathione (GSH) to glutathione disulfide (GSSG) by glutathione peroxidase (Fig. 6c).

Our data show that levels of the oxidized form (GSSG) were higher in (Tg/Tg) and (Tg/+) fish than those in (+/+) and (+/Tg) fish, whereas levels of the reduced form (GSH) were lowest in (Tg/Tg) fish (Fig. 6d and “Supplementary Information”). However, these data could not be statistically interpreted owing to the lack of repetition of the experiment.

## Discussion

Previously, we reported that short and monounsaturated fatty acid levels are significantly reduced in GH-transgenic amago salmon, indicative of increased energy production via β-oxidation^18^. Our iTRAQ data showed increased levels of complex I component proteins (e.g., NADH-ubiquinone oxidoreductase chains 1, 2, and 5), III, and IV, with increased ATP synthase expression in (Tg/Tg) fish (Table 1 and Supplementary Table 1). Although these results indicate that (Tg/Tg) fish exhibit enhanced electron transport using NADH produced from the TCA cycle, ATP concentration was markedly decreased compared with that in (+/+) fish. These contradictory results suggest that (Tg/Tg) fish exhibit mitochondrial dysfunction.

Furthermore, we observed a significant decrease in the NAD^+^/NADH ratio in female transgenic fish (Fig. 3b). The NAD^+^/NADH ratio indicates the overall status of mitochondrial metabolism^21,22^, and a decreased ratio results in increased PGC-1α expression via thioredoxin interacting protein (TXNIP)^23^. PGC-1α is involved in mitochondrial biogenesis, respiration, thermogenesis, and gluconeogenesis^24^ and acts as a potent regulator of ROS removal via upregulation of various ROS-detoxifying enzymes^25^. Multiple pathways can stimulate PGC-1α in response to low NAD^+^ levels, and there is crosstalk between PGC-1α and OPA1. The signal transduction pathway between them is complex and involves both stimulation and downregulation, rather than only the conversion of PGC-1α to OPA1^26^. At least 21 pathways exist from PGC-1α to OPA1, and 29 pathways from OPA1 to PGC-1α (Fig. 3c, Supplementary Table 3). OPA1 is localized on the inner mitochondrial membrane and regulates the equilibrium between mitochondrial fusion and fission and is, therefore, essential for maintaining mitochondrial function^27^. As ATP production was found to be associated with OPA1^26,28,29^, increased OPA1 expression in (Tg/Tg) fish indicates an increase in ATP synthase expression and has a central role in the coordinated regulation of mitochondrial structure and function^26^. Notably, there were differences in the five protein signals corresponding to OPA1 in mice^27^. (Tg/Tg) fish showed a different intensity band signal than (+/+) fish. The exact banding pattern was difficult to determine due to the strong band intensity of (Tg/Tg) fish (“Supplementary Information”). The reason for this needs to be investigated in the future. Thus, mitochondrial fusion may compensate for the shortage of ATP resulting from dysfunctional mitochondria (Fig. 2e,f).

Upregulation of PGC-1α and OPA1 is observed in hypoglycemia (Fig. 3c)^28,30^, consistent with previous data (Fig. 3a)^18^ in terms of enhancing β-oxidation and promoting the TCA cycle for ATP production. Therefore, a decreased NAD^+^/NADH ratio is predicted to be the key regulator of this phenomenon [observed in (Tg/Tg) and (Tg/+) GH-transgenic fish]. Moreover, this phenomenon is inherited by the future generations of female GH-transgenic fish and male offspring, who will not pass it on to their offspring. If this hypothesis is correct, a low NAD^+^/NADH ratio will increase the expression of PGC-1α and OPA1 in these two types of fish. Inducing the expression of these genes leads to TCA cycle activation, resulting in hypoglycemia and decreased ROS levels in (Tg/Tg) and (Tg/+) fish. Indeed, blood glucose levels were significantly decreased in these transgenic fish (Fig. 1b,c). Collectively, our results indicate a dysfunctional *ND1* site.

As the loss of mtDNA-encoded *ND1* results in the disruption of complex I^31^, it also affects the activities of other respiratory chains, resulting in mitochondrial dysfunction. Therefore, (Tg/Tg) and (Tg/+) fish may be unable to form complex I. We speculated that the reduced function of complex I may be responsible for the reduced NAD^+^/NADH ratio and ATP production, which would result in reduced ROS levels in (Tg/Tg) and (Tg/+) fish, as summarized in Fig. 3a,c. Indeed, ROS levels were decreased in these fish (Fig. 5d); the inferred metabolic flow of (Tg/Tg) is shown in Fig. 3a.

Mutations in mitochondrial genes profoundly affect cellular respiration and result in the development of mitochondrial diseases^32^. Such mutations include single base substitutions (point mutations), base deletions, and gene duplications^33^ (direct duplications). NADH-ubiquinone oxidoreductase (complex I)—located in the inner mitochondrial membrane—is an energy-metabolizing enzyme that catalyzes the first reaction of the respiratory chain, resulting in ATP synthesis^34^. Complex I consists of 41 subunits, 7 of which are encoded by the mtDNA-encoded genes^35^; *ND1* is one of these seven subunits. Several genetic mutations in *ND1* have been reported in humans and are associated with diseases, including Leber hereditary optic neuropathy, mitochondrial encephalopathy with lactic acidosis and stroke-like episodes syndrome, diabetes mellitus, non-insulin-dependent, mitochondrial complex I deficiency, and Alzheimer’s disease (https://www.uniprot.org/uniprot/P03886). Deletion mutations in mtDNA have been reported in 1,312 cases of mitochondrial diseases and account for at least 20% of all cases^36^. Of the reported mtDNA deletion mutations, approximately 90% occur in *ND4*^32^. In contrast, with only three cases reported to date, the number of deletion mutations in *ND1* is extremely low^32,36^.

It remains unclear why the novel deletion in *ND1* is only present in (Tg/Tg) and (Tg/+) fish. However, mitochondrial genes exhibit a tenfold higher mutation rate than nuclear genes^37^. Although mtDNA repair systems exist, they are not sufficient to offset the ROS levels generated via oxidative phosphorylation. This can be attributed to the proximity of mtDNA to the ROS^38^ generation sites in the inner mitochondrial membrane^39^. Thus, a spontaneous mutation in mtDNA may occur in response to increased ROS levels associated with excess energy production in the GH-transgenic amago salmon.

GH stimulates catabolic reactions and enhances oxidative stress in GH-transgenic rat ^40^. Catalase (CAT), an enzyme involved in removing ROS, showed decreased catalase activity protein and mRNA expression in the liver and kidney of GH transgenic mice compared to that in wild-type mice. However, these were increased in GH-deficient Ames dwarf mice compared to wild-type mice ^41^. In the case of fish, GH-transgenic zebrafish exhibited higher oxygen consumption and ROS production than homo, hetero, and wild-type fish, in that order^42^. GH transgenic coho salmon also showed higher protein carbonyl, an indicator of protein oxidation by free radicals, and lower antioxidant enzyme activity and GSH levels compared with those of wild-type fish^43^. Conversely, the glutathione content was higher in the liver, muscle, and plasma of GH-transgenic coho salmon fed a satiating diet than in wild-type fish^44^. Diet has a greatly effect on the metabolism of GH transgenic fish. For example, GH transgenic common carp (*Cyprinus carpio*) fed high dietary protein had better growth performance than non-transgenic common carp, lower glycolysis and lipid synthesis, and increased fatty acid oxidation^45^. In contrast, increasing dietary starch in this fish promoted glycolysis and lipid synthesis and suppressed glycogenesis and fatty acid β-oxidation^46^. Thus, it can be predicted that ROS production varies significantly due to the metabolic changes caused by diet. Generally, high ROS has been reported in GH transgenic animals due to the effect of GH, and there are few reports of low ROS as in the case of GH-transgenic amago salmon.

Furthermore, iTRAQ revealed that several ROS scavenging systems, including superoxide dismutase, peroxiredoxin, and thioredoxin, were upregulated in (Tg/Tg) fish (Supplementary Table 1), compared with those in (+/+) fish. The overexpression of A long-chain-fatty-acid-CoA ligase 1 (ACSL1) compensates for mitochondrial dysfunction and protects cells from oxidative stress and cellular injury^47^, and glucose-regulated protein 78 kDa (GRP78) reduces the net flux of Ca^2+^ from the endoplasmic reticulum to mitochondria and reduces free radical production^48^. This suggests that high expression of GRP78 and ASCL1, by hypoglycemia and decreased fatty acid synthesis, respectively, may also occur in (Tg/+) fish. Furthermore, glucose deprivation increases GSSG levels^49^; therefore, a trend toward elevated GSSG levels is expected in (Tg/Tg) and (Tg/+) fish, which exhibit low serum glucose levels (Fig. 6d). The GSSG/GSH ratio is an indicator of oxidative stress and ROS production^50^; in (Tg/Tg) and (Tg/+) fish, this ratio exceeded 1.0. This is indicative of higher ROS production in (Tg/Tg) and (Tg/+) fish than that in (+/+) and (+/Tg) fish. As shown in the results, these were analyzed using pooled data. In general, GH-transgenic fish tend to have much greater variability in the measured variables than non-transgenic fish; therefore, analyses using more experimental fish are necessary.

We speculated that large amounts of ROS might have been generated in (Tg/Tg) and (Tg/+) fish owing to *ND1*-deletion-induced mitochondrial dysfunction; however, these ROS species were immediately eliminated by the scavenging systems. In particular, the first female fish produced by transgenesis appeared to possess a greater amount of ROS than male GH-transgenic or non-transgenic fish. This may subsequently exert pressure on its ROS scavenging systems controlled by cellular antioxidant defense systems^51^. Moreover, ROS production is initiated in response to superoxide generation by NADPH oxidase, one of the major sources of ROS^52^ involved in regulating cell signaling, inflammation, cell growth, and death^53^. Consequently, levels of NADPH oxidase also tended to decrease. Therefore, this assumption appears to be plausible because the ROS levels in (Tg/Tg) and (Tg/+) fish are lower than those in (+/+) and (+/Tg) fish (Fig. 5d).

Therefore, factors such as GH-induced production of ROS during growth enhancement and transgenesis, particularly for successful transgenesis in females, may lead to novel mtDNA deletion mutations, such as the *ND1* deletion reported in this study, that continue to be maternally inherited across generations. Here, we were unable to elucidate why female GH-transgenic fish exhibited a novel deletion in *ND1*. Even individuals who would not usually survive can inherit pathological traits in the next generation due to genetic modification or additional factors. However, genetically mutated fish are not usually selected as breeding stock owing to their reduced performance.

Further research on the effects of manipulating genes other than those encoded by the nuclear genome will clarify the associated mechanisms. Furthermore, as a growth-enhanced transgenic line of salmon has been approved for human consumption. There is need to elucidate genetic and metabolic characteristics in salmon to improve aquaculture practices.

## Materials and methods

### Experimental animals and samples

GH-transgenic amago salmon was produced by microinjection of a plasmid harboring On*MTGH1*, a sockeye salmon *GH1* gene fused with metallothionein-B promoter^7^, into fertilized eggs of amago salmon. Homozygous and hemizygous GH-transgenic amago salmon were produced as per the method described in a previous study^18^. F1 transgenic (Tg/+) and (+/+) fish were produced by fertilizing one female mosaic egg with wild sperm (+/+). These F1 fry were reared until maturity, and F1 (Tg/+) fish were selected by PCR using the *OnMTGH1* sequencing primer. To confirm that these selected F1s were F1(Tg/+), they were crossed with wild-type (+/+) fish. If F1 is (Tg/+), then the ratio of (Tg/+) to (+/+) will be 1:1. This selection was performed by PCR using *OnMTGH1* sequencing primers^18^. After selecting only those fish whose F1 was (Tg/+), these F1 (Tg/+) fish were fertilized with each other to produce (Tg/Tg) fish. In this fertilization, approximately 25% and 50% of the fish were expected to be homozygous (Tg/Tg) and hemizygous, respectively. As the (Tg/Tg) GH gene inserted is twice as much as that in hemizygotes, only homozygotes (Tg/Tg) were selected by real-time PCR. The homozygous (Tg/Tg) fish was maintained by fertilizing (Tg/Tg) eggs with (Tg/Tg) sperm.

Subsequently, (Tg/+) was produced by fertilizing a (Tg/Tg) female with a wild-type male (+/+). Only fish confirmed by real-time PCR to produce 100% (Tg/+) in this cross were used in the experiment. In addition, (+/Tg) was produced by fertilizing wild-type females (+/+) with (Tg/Tg) males. In a similar manner, only fish with 100% (+/Tg) confirmed by real-time PCR were selected.

In this manner, (Tg/+) and (+/Tg) fish were produced every year by crossing (Tg/Tg) and wild-type (+/+) males or females. The fish used in the experiment were 4–5 months of age.

These GH-transgenic fish were maintained at the National Research Institute for Marinated Aquaculture, Fisheries Research Center (Tamaki, Japan). The test fish were reared in a circulating tank at 15 °C under natural light and dark phases of similar intensity. Feed was provided to the fish up to 3 d before sampling.

The fish were euthanized under anesthesia (200 μL/L of 2-phenoxyethanol), and then the dissected livers were rapidly frozen in liquid nitrogen and stored at − 80 °C until further use. F5 transgenic amago salmon were used in this experiment.

### Measurement of blood glucose and d-ROM test

Blood samples were collected using a 20-gauge needle and then centrifuged for 10 min at 9200×*g*. The serum was immediately frozen in liquid nitrogen. Glucose concentration in the serum was measured using Fuji Dry Chem 3030 (Fujifilm, Tokyo, Japan). Oxidative stress in the serum was determined via a d-ROMs test (WISMERLL, Tokyo, Japan), according to the manufacturer’s protocol. The statistical data are described in the “Supplementary Methods”.

### iTRAQ-based proteomic analysis

After dissection, the liver tissues of (Tg/Tg) and (+/+) fish were removed and immediately placed in liquid nitrogen. Eighty micrograms of proteins were extracted from the liver tissues of (+/+) and (Tg/Tg) fish (114:116 = +/+; 115:117 = Tg/Tg), followed by labeling of each protein solution with iTRAQ reagent 4 plex (AB Sciex, Redwood City, CA, USA) for iTRAQ-based proteomic analysis. The liver tissues were used without pooling. The detailed protocol is described in the “Supplementary Methods”.

### LC–MS analysis of NAD^+^/NADH

Frozen liver tissues (*n* = 3) obtained from four types of fish (Tg/Tg, Tg/+,+/Tg, +/+) were weighed (~ 15 mg) in a 2.0-mL microcentrifuge tube and analyzed without pooling. The detailed protocol is described in the “Supplementary Methods”.

### IPA

The IPA protocol has been previously described in detail^54^ and is described in the “Supplementary Methods”. In addition, the relationships between phenotype and NADPH oxidase were predicted as previously described^18^.

### Western blotting

iTRAQ-based proteomic data were confirmed using western blotting. A reducing agent (DTT: 30 mg/500 μL) was added to each protein fraction, after which the samples were denatured at 98 °C for 5 min. Proteins were separated by sodium dodecyl sulfate–polyacrylamide gel electrophoresis (100 V and 500 mA for 90 min) and then transferred (100 V and 500 mA for 1 h) onto a polyvinylidene difluoride (PVDF) membrane, which was then blocked (5% skim milk) for 1 h and incubated overnight at 4 °C with primary antibodies (see “Supplementary Methods”). After primary antibody incubation, the PVDF membrane was washed with 1 × TBST (thrice, 5 min each) and incubated with the secondary antibody (rabbit IgG/mouse IgG; 1:1000; #7074; Cell Signaling Technology, Danvers, MA. USA) for 1 h. The PVDF membrane was then washed, and the protein of interest was visualized via chemiluminescence after adding a horseradish-conjugated substrate. Semi-quantitative analysis of protein expression was performed using Image J (NIH, Bethesda, MD, USA).

### CE-TOF–MS analysis of liver tissues

Liver tissues from five GH homozygous and hemizygous fish and non-transgenic siblings were excised and immediately frozen in liquid nitrogen. Thereafter, frozen liver tissue (200 mg) was homogenized in ethanol and pooled as one sample for CE-TOF–MS analysis. CE-MS analysis of liver tissues was performed as previously described^55^. The detailed protocol can be found in the “Supplementary Methods”.

### Immunofluorescence

Frozen liver tissues were sectioned (control, homozygous sample number, *n* = 3) into 10-µm sections using a CM 1950 cryostat (Leica Microsystems, Wetzlar, Germany) and fixed with 4% paraformaldehyde (Nacalai Tesque, Tokyo, Japan) for 20 min. The sections were washed (thrice, 5 min each) with 1 × PBS and incubated with a blocking reagent (Blocking One Histo, Nacalai Tesque) for 20 min at 25 °C. After washing with 1 × PBS (thrice, 5 min each), the sections were incubated overnight with anti-ATP5a (1:100; Abcam, Cambridge, UK) at 4 °C, followed by washing with 1 × PBS (thrice, 5 min each) and incubating with anti-rabbit IgG Alexa 488 (1:1000; Abcam) for 2 h at 25 °C. The sections were washed with 1 × PBS again (thrice, 5 min each) and incubated with a drop of 4′,6-diamidino-2-phenylindole (DAPI) for 20 min at 25 °C, followed by confocal laser microscopy (FV1000 D; Olympus, Tokyo, Japan). The obtained data were analyzed using image analysis software (Photoshop; Adobe, Mountain View, CA, USA), and the total mitochondrial area per cell was calculated.

### TEM

Three different GH homozygous fish and non-transgenic siblings were used for thin section analysis. The excised liver was fixed overnight with 2.5% glutaraldehyde prepared in 0.15-M cacodylic buffer (pH 7.4). Subsequently, the fixed tissue was incubated with 1% osmium tetroxide prepared in 0.15 M cacodylic buffer (pH 7.4) for 2 h, after which the tissue was dehydrated with ethanol. The dehydrated samples were embedded in epoxy resin Quetol-812 (Nisshin EM Co. Ltd., Tokyo, Japan).

Ultra-thin sections were prepared by sectioning the embedded samples using an ultramicrotome (ULTRA CUT UCT; Leica Microsystems). The sections were observed by TEM (JEM-1200EX; JEOL, Tokyo, Japan). The obtained data were used to calculate the mitochondrial area per mitochondrion using ImageJ.

### Mitochondrial genome analysis, genome assembly, variant analysis, validation of SNPs, and deletion mutation analysis

Mitochondrial genetic analysis was performed separately using two different (+/+) and (Tg/Tg) fish. As mutations were evident, three more (+/+), (Tg/Tg), (Tg/+), and (+/Tg) fish were added for further *ND1* site analysis. These data were obtained from independent fish, and samples were not pooled. For mutation analysis, the following software were used: SolexaQA v3.1.7.1, BWA v0.7.15, Picard v1.119, and GATK v3.8.0. Mini seq for *ND1* was performed using BWA v0.7.17, and GATK v4.0.1.0 and v4.0.5.0. All software parameters were set to default. Details of these protocols are provided in the “Supplementary Methods”.

### RNA extraction for Illumina analysis

One fish from the (Tg/Tg) group was used for RNA-seq. RNA was extracted from liver tissues (0.5 mg) using the RNeasy Mini Kit (Qiagen, Hilden, Germany), as per the manufacturer’s instructions. Purified total RNA was electrophoresed on a 2.0% agarose gel to assess quality. RNA was quantified using a Bio photometer (D30, Eppendorf, Hamburg, Germany). The extracted samples were used for RNA Illumina analysis. For mutation analysis using RNA seq, AfterQC v0.9.6 and Hisat2 v2.1.0 were used; all software parameters were set to default.

### Immunoprecipitation

The Dynabeads™ Protein G for Immunoprecipitation kit (Thermo Fisher Scientific, Waltham, MA, USA) was used for immunoprecipitation with anti-*ND1* (#sc-293243; Santa Cruz Biotechnology Inc, Dallas, TX, USA). Proteins were purified as per the manufacturer’s instructions.

### Statistical analysis

Data are expressed as the mean ± SE. The significance of western blot and mitochondrial area data was determined using the Student’s *t*-test, while that of serum glucose data was determined by one-way analysis of variance (ANOVA) followed by a posteriori comparison of the significant ANOVA results using Bonferroni or Dunnett’s T3 test. Levene’s multiple comparison test for variances indicated that the assumption of homogeneity of variances was rejected at the 5% significance level. For statistical decisions, Dunnett’s T3 multiple comparison test, which is robust in testing means under heterogeneous variance, was used to evaluate means; *p* values < 0.05 were considered statistically significant.

### Ethical statement

All animal experiments were conducted by trained personnel in accordance with the Animal Care Committee guidelines of Nihon University. The authors declare that this manuscript complies with the Nature Ethical Guidelines for Journal Publication. Nihon University. Ethical approval for the use of animals was given by the National Research Institute of Aquaculture, Fisheries Research and Education Agency Ethics Committee (#29013). We performed this study in accordance with the ARRIVE guidelines (https://arriveguidelines.org).

## Supplementary Information

Supplementary Tables.

Supplementary Figures.

Supplementary Information.

## Data Availability

The sequencing data have been deposited in the DDBJ BioProject database under the accession numbers DRR298832-DRR298846.
