# Supplementary material for: A novel ND1 mitochondrial DNA mutation is maternally inherited in growth hormone transgenesis in amago salmon (Oncorhynchus masou ishikawae)
Source: Sci Rep. 2022 Apr 25;12:6720. doi: 10.1038/s41598-022-10521-4 (PMC9038734; doi:10.1038/s41598-022-10521-4)
Supplement: Supplementary file 1 — Supplementary Tables. [file 41598_2022_10521_MOESM1_ESM.pdf]

Supplementary Table 1. List of 270 proteins with ≥ 1.5-fold increase in expression as determined by iTRAQ.

No.	Accession no.	Protein name	Fold change Homo/control
1	Q92109	Cytochrome P450 1A3	7.727
2	P86979	Fructose-bisphosphate aldolase A (Fragment)	6.711
3	Q92148	Cytochrome P450 1A1	6.030
4	Q92116	Cytochrome P450 1A1	5.381
5	Q8JFQ6	Keratin, type I cytoskeletal 13	4.808
6	C0HJT7	Hemoglobin subunit beta-2	4.245
7	Q92110	Cytochrome P450 1A1	4.226
8	Q1AGS7	Hemoglobin subunit beta-1	3.735
9	P02142	Hemoglobin subunit beta-1	3.462
10	P83964	Natriuretic peptides A	3.401
11	O93297	Cytochrome P450 2K4	3.346
12	P32847	Band 3 anion exchange protein	3.325
13	B9EMY6	Flap endonuclease 1	3.246
14	P98093	Complement C3 (Fragment)	3.203
15	Q98SN8	Protein transport protein Sec61 subunit alpha isoform B	3.042
16	P84204	Hemoglobin cathodic subunit beta	3.032
17	P56251	Hemoglobin subunit beta	3.005
18	P02141	Hemoglobin subunit beta-4	2.999
19	Q91487	60S ribosomal protein L13a (Fragment)	2.861
20	P86810	L-amino-acid oxidase	2.856
21	P02019	Hemoglobin subunit alpha-1	2.850
22	P45721	Hemoglobin subunit beta-C	2.838
23	O42563	Cytochrome P450 3A27	2.804
24	O57561	Heat-shock 70 kDa protein 1	2.802
25	Q90X85	Complement component C8 beta chain	2.771
26	Q9ZZM3	NADH-ubiquinone oxidoreductase chain 5	2.758
27	Q90YU9	60S ribosomal protein L18a	2.729
28	Q90YW0	60S ribosomal protein L9	2.721
29	Q90YT5	60S ribosomal protein L34	2.718
30	P25489	Sodium/potassium-transporting ATPase subunit alpha-1	2.716
31	P27446	Tyrosine-protein kinase Fyn	2.706
32	P80946	Hemoglobin anodic subunit beta	2.688
33	P49843	Glucocorticoid receptor	2.671
34	C1BK83	Nucleoporin seh1	2.667
35	P35032	Trypsin-2 (Fragment)	2.587
36	Q4SS66	RISC-loading complex subunit tarbp2	2.579
37	P28270	Glutamate dehydrogenase (Fragments)	2.545
38	Q90YR6	40S ribosomal protein S8	2.515
39	P06682	Complement component C9 (Fragment)	2.483
40	Q90YT3	60S ribosomal protein L35a	2.482
41	Q98TS0	Hemoglobin subunit beta	2.475
42	O78682	Cytochrome c oxidase subunit 2	2.472
43	Q9I8F9	Heat-shock 70 kDa protein 1	2.471
44	B5XB27	Kynurenine formamidase	2.470
45	P49947	Ferritin, middle subunit	2.467
46	Q90508	Vitellogenin-1	2.463
47	Q90YS0	40S ribosomal protein S4	2.459
48	P69090	60S ribosomal protein L18	2.455
49	Q9YGF2	40S ribosomal protein S6	2.448
50	Q90YU5	60S ribosomal protein L23	2.430
51	Q92087	Aromatase	2.420
52	P61367	60S ribosomal protein L15	2.415
53	P85835	Ferritin, middle subunit	2.402
54	O42446	Deoxyribonuclease-1	2.401
55	P82264	Glutamate dehydrogenase, mitochondrial	2.381
56	B5X3X5	Katanin p60 ATPase-containing subunit A1	2.375
57	Q98893	Vitellogenin-2	2.371
58	P79896	Alcohol dehydrogenase class-3	2.370
59	P87383	Guanine nucleotide-binding protein G(i) subunit alpha-1	2.361
60	P18288	Tubulin alpha chain, testis-specific	2.359
61	P24558	60S ribosomal protein L18	2.354
62	Q9W6X9	40S ribosomal protein S24	2.347
63	Q9YIC0	Elongation factor 1-alpha	2.344
64	A6P7L8	Piwi-like protein 2	2.341
65	B5XB24	ER membrane protein complex subunit 4	2.330
66	P14527	Hemoglobin subunit alpha-4	2.330
67	P24774	Retinol-binding protein 4-A	2.328
68	Q90YS2	40S ribosomal protein S3	2.325
69	Q90YQ1	40S ribosomal protein S23	2.324
70	Q90WG6	N-acylneuraminate cytidylyltransferase	2.324
71	P48171	Cytochrome c oxidase subunit 2	2.323
72	O42249	Guanine nucleotide-binding protein subunit beta-2-like 1	2.321
73	Q7ZZX9	GTP-binding nuclear protein Ran	2.292
74	Q90327	Mitogen-activated protein kinase 8A	2.277
75	P30436	Tubulin alpha chain	2.274
76	P28770	Ependymin-1	2.258
77	P58312	Sodium/potassium-transporting ATPase subunit alpha-3	2.252
78	Q90YW1	60S ribosomal protein L8	2.249
79	Q90YV5	60S ribosomal protein L13	2.248
80	Q91483	Parvalbumin beta 2	2.247
81	Q9W644	Proliferating cell nuclear antigen	2.245
82	Q91061	Ictacalcin	2.239
83	Q90YT1	60S ribosomal protein L37	2.235
84	P83270	Hemoglobin subunit alpha-1	2.231
85	P70083	Sarcoplasmic/endoplasmic reticulum calcium ATPase 1	2.231
86	P48175	NADH-ubiquinone oxidoreductase chain 2	2.222
87	P70066	40S ribosomal protein S15	2.214
88	B5RI54	Eukaryotic translation initiation factor 3 subunit H	2.211
89	O42294	Rhodopsin (Fragment)	2.206
90	Q90YV7	60S ribosomal protein L11	2.196
91	P61359	60S ribosomal protein L27	2.190
92	Q60FY0	G2/mitotic-specific cyclin-B1	2.187
93	P68528	ATP synthase protein 8	2.186
94	Q90YQ8	40S ribosomal protein S15a	2.183
95	O48358	NADH-ubiquinone oxidoreductase chain 1	2.181
96	Q90YC0	Elongation factor 1-gamma	2.180
97	Q9PW88	G-protein coupled receptor family C group 6 member A	2.160
98	P81018	Ladderlectin	2.158
99	B5DGM7	Fructose-bisphosphate aldolase A	2.157
100	Q90YS3	40S ribosomal protein S2	2.150
101	P58372	60S ribosomal protein L30	2.149
102	P61487	60S ribosomal protein L36a	2.144
103	Q90YV8	60S ribosomal protein L10a	2.143
104	Q9YHC3	Tubulin beta-1 chain	2.142
105	Q804X9	Melanopsin-A	2.140
106	B5DG42	Eukaryotic initiation factor 4A-III	2.136
107	Q90YR7	40S ribosomal protein S7	2.127
108	P85292	Nucleoside diphosphate kinase B (Fragments)	2.124
109	O93428	Cathepsin D	2.124
110	Q2KN95	Cytospin-A	2.119
111	B5XD90	LYR motif-containing protein 4	2.106
112	P31579	Low choriolytic enzyme	2.100
113	Q8AY73	Exportin-2	2.092
114	Q90YU8	60S ribosomal protein L19	2.090
115	Q5DVH6	60S ribosomal protein L35	2.071
116	Q90341	Transcriptional regulator Myc-1	2.066
117	P81459	Cytochrome c	2.066
118	Q7SYF8	Keratin, type I cytoskeletal 18	2.064
119	Q6Y263	60S ribosomal protein L24	2.064
120	Q9DGI3	Thioredoxin	2.056
121	Q90339	Myosin heavy chain, fast skeletal muscle	2.053
122	B5X4E0	Calumenin-B	2.052
123	P49055	Actin, alpha skeletal muscle	2.047
124	Q9PT08	Small ubiquitin-related modifier 1	2.046
125	Q90YX1	60S acidic ribosomal protein P0	2.041
126	Q9W6X3	10 kDa heat shock protein, mitochondrial	2.040
127	P83751	Actin, cytoplasmic 1	2.038
151	Q91240	Tubulin beta chain	1.946
152	B5XCA1	Adenylate kinase 2, mitochondrial	1.945
153	Q19A30	Aldehyde dehydrogenase family 9 member A1	1.941
154	P80977	Cytochrome c oxidase subunit 6C-1	1.941
155	P61155	40S ribosomal protein S19	1.940
156	O93248	Dynamin-like 120 kDa protein, mitochondrial	1.938
157	O73683	Amyloid beta A4 protein	1.936
158	Q9PRF8	Beta-2-microglobulin	1.935
159	B5X2S3	Actin-related protein 8	1.927
160	O42161	Actin, cytoplasmic 1	1.922
161	B5X165	Protein SMG9	1.920
162	P21848	Serum albumin 1	1.918
163	P07746	High mobility group-T protein	1.917
164	C0HBB2	Glycosylated lysosomal membrane protein	1.909
165	Q90YQ2	40S ribosomal protein S21	1.900
166	C1BJ98	Ubiquitin-fold modifier 1	1.899
167	P32759	Alpha-1-antitrypsin homolog	1.897
168	P11251	Hemoglobin subunit alpha	1.892
169	P54863	Somatolactin-1	1.891
170	Q90YT7	60S ribosomal protein L31	1.888
171	Q804S2	Bone morphogenetic protein 2	1.887
172	B5X4Y9	Phosphotriesterase-related protein	1.882
173	P18520	Intermediate filament protein ON3	1.880
174	Q9T9J2	Cytochrome b	1.876
175	B6VA23	Serine/threonine-protein phosphatase 2A 55 kDa regulatory subunit B beta isoform	1.871
176	P86177	L-rhamnose-binding lectin CSL1	1.869
177	P19618	NADPH-cytochrome P450 reductase (Fragments)	1.867

128	C0HJT8	Hemoglobin subunit alpha (Fragment)	2.035
129	Q6Y1Z4	SUMO-conjugating enzyme UBC9	2.033
130	B5DGI7	RNA-binding protein 8A	2.032
131	P08108	Heat shock cognate 70 kDa protein	2.031
132	B5DGL6	40S ribosomal protein S3a	2.021
133	O13127	Flotillin-1	2.019
134	B5X3Z6	Lissencephaly-1 homolog A	2.017
135	Q90YW2	60S ribosomal protein L7a	2.016
136	B5X9P2	Probable cytosolic iron-sulfur protein assembly protein ciao1-A	2.015
137	P80971	Cytochrome c oxidase subunit 4 isoform 2, mitochondria	2.009
138	Q6S9V6	Citrate synthase, mitochondrial	2.003
139	Q9DGM7	Major vault protein (Fragment)	1.998
140	P52865	60S ribosomal protein L22 (Fragment)	1.994
141	O13085	Cytochrome c oxidase subunit 6A, mitochondrial	1.980
142	B5DGB6	40S ribosomal protein SA	1.979
143	P68200	Ubiquitin-40S ribosomal protein S27a	1.971
144	Q90YT4	60S ribosomal protein L35	1.970
145	P21919	Somatolactin	1.965
146	B5X5B4	Elongation factor Ts, mitochondrial	1.965
147	P26351	Thymosin beta-11	1.956
148	B9EPI1	CDGSH iron-sulfur domain-containing protein 2A	1.950
149	P34205	Deoxyribodipyrimidine photo-lyase	1.950
150	Q9W6Y0	40S ribosomal protein S30	1.949
178	O42259	Glyceraldehyde-3-phosphate dehydrogenase	1.866
179	Q6UFZ9	14-3-3 protein beta/alpha-1	1.863
180	C1BXU5	Ubiquitin-like protein 4A	1.863
181	Q9DFR6	40S ribosomal protein S13	1.855
182	B5X277	Methylthioribulose-1-phosphate dehydratase	1.855
183	B5XGH3	Transcription and mRNA export factor ENY2-2	1.847
184	P02719	Sodium channel protein	1.847
185	P84231	Histone H3.2	1.842
186	Q4RSW7	Glutamyl-tRNA(Gln) amidotransferase subunit C, mitochondrial	1.838
187	Q6UFZ8	14-3-3 protein beta/alpha-2	1.828
188	B9ENE7	Protein mago nashi homolog	1.827
189	B5X3W7	Choline transporter-like protein 2	1.825
190	Q90YT0	60S ribosomal protein L37a	1.824
191	P51467	Arrestin red cell isoform 2	1.816
192	P62797	Histone H4	1.815
193	Q90YT6	60S ribosomal protein L32	1.815
194	P48673	Vimentin beta	1.813
195	P83456	Alkaline phosphatase	1.812
196	Q6UZG0	40S ribosomal protein S29	1.812
197	Q9PTY0	ATP synthase subunit beta, mitochondrial	1.809
198	Q90YQ7	40S ribosomal protein S16	1.805
199	B5DG67	Ribosome biogenesis protein wdr12	1.804
200	Q90YQ4	40S ribosomal protein S19	1.803

No.	Accession no.	Protein name	Fold change Homo/control
201	Q90YT2	60S ribosomal protein L36	1.802
202	B5X3C1	Galactocerebrosidase	1.799
203	Q9YGL2	L-lactate dehydrogenase B chain	1.797
204	P55202	Atrial natriuretic peptide receptor 2	1.795
205	Q4SG99	Cytochrome c	1.795
206	P02594	Calmodulin	1.791
207	P69069	Histone H2B	1.788
208	P24722	Creatine kinase, testis isozyme	1.783
209	Q90YQ5	40S ribosomal protein S18	1.783
210	P22647	Histone H2A.Z	1.780
211	Q90YR4	40S ribosomal protein S10	1.778
212	P45433	Translocon-associated protein subunit alpha	1.775
213	Q9PTW9	Proteasome subunit alpha type-7	1.771
214	P80856	Fatty acid-binding protein, liver	1.758
215	Q90YU6	60S ribosomal protein L22	1.753
216	O42305	Flotillin-2	1.748
217	P28771	Ependymin-2	1.747
218	C0HBG1	Anamorsin-B	1.746
219	E7FKV8	Polycystic kidney disease 1 like 1	1.744
220	Q9YH37	Ras-related protein Rap-1b	1.742
221	Q91195	Cystatin	1.739
222	P84652	Hemoglobin subunit beta	1.738
223	P05774	Ras-like protein (Fragment)	1.736
224	B5X0W9	Isochorismatase domain-containing protein 1	1.725
225	Q90YQ6	40S ribosomal protein S17	1.722
226	P56533	Betaine aldehyde dehydrogenase	1.722
227	P0DM59	Bilirubin-inducible fluorescent protein UnaG	1.722
228	O13019	40S ribosomal protein S12	1.720
229	Q03156	Serum albumin 2	1.719
230	O57607	Keratin, type I cytoskeletal 18	1.711
231	Q90YP9	40S ribosomal protein S25	1.706
232	Q91453	Stonustoxin subunit beta	1.705
233	P53447	Fructose-bisphosphate aldolase B	1.704
234	P84335	Tropomyosin alpha-1 chain	1.702
235	Q91191	Peroxiredoxin	1.701
236	B5X9S3	Coiled-coil domain-containing protein 58	1.699
237	P03946	Superoxide dismutase [Cu-Zn]	1.692
238	O57523	Apolipoprotein A-I-1	1.681
239	O73817	Proteasome subunit beta type-3	1.676
240	P22642	Ventricular natriuretic peptide	1.675
241	Q9PW98	Gonadotropin subunit beta-2	1.668
242	Q9W725	Mitochondrial uncoupling protein 2	1.667
243	B5X370	Toll-interacting protein	1.662
244	Q202I6	Translationally-controlled tumor protein homolog	1.660
245	P58165	Plasma membrane calcium-transporting ATPase 2 (Fragment)	1.646
246	C3KHF2	Ubiquitin-like protein 4A	1.636
247	P51596	GTP cyclohydrolase 1 (Fragment)	1.630
248	Q9YGP3	Glycoprotein hormones alpha chain	1.628
249	Q9IA78	Transcription initiation factor IIA subunit 2	1.624
250	P48251	Gonadotropin subunit beta-2	1.608
251	P80334	Cytochrome c oxidase polypeptide VIIc (Fragment)	1.608
252	P83856	Kininogen (Fragments)	1.607
253	O73672	Proteasome subunit alpha type-2	1.602
254	Q9I954	Thymosin beta-b	1.601
255	P80081	6-pyruvoyl tetrahydrobiopterin synthase (Fragments)	1.599
256	O13008	Fatty acid-binding protein, heart	1.597
257	Q8AWF2	Nascent polypeptide-associated complex subunit alpha	1.589
258	Q589R5	Triosephosphate isomerase	1.583
259	P00026	Cytochrome c iso-1/iso-2	1.582
260	P06350	Histone H1	1.581
261	P80973	Cytochrome c oxidase subunit 5A-2, mitochondrial (Fragment)	1.560
262	Q90YP3	40S ribosomal protein S28	1.555
263	P49253	Amine oxidase [flavin-containing]	1.549
264	B5DGQ7	Beta-enolase	1.536
265	B5X186	Calumenin-A	1.532
266	Q9YH26	Sodium/potassium-transporting ATPase subunit alpha-1	1.522
267	Q8JFR1	Lysozyme g	1.515
268	P81036	Superoxide dismutase [Cu-Zn] (Fragment)	1.505
269	P27007	Apolipoprotein A-I	1.503
270	Q92038	Acyl-CoA desaturase	0.355

Supplementary Table 2: Signal transduction networks analyzed based on iTRAQ data using IPA
"ND" indicates that the Z-score could not be calculated, such as “Z-Score=0” or “no active pattern available”.
© 2000-2021 QIAGEN. All rights reserved.

Ingenuity Canonical Pathways	-log(p-value)	Ratio	z-score	Molecules
EIF2 Signaling	56.9	0.232	4.899	ACTA1,ACTB,EIF3H,EIF4A3,FAU,RAP1B,RPL10A,RPL11,RPL13,RPL13A,RPL18,RPL18A,RPL19,RPL22,RPL24,RPL27,RPL31,RPL32,RPL34,RPL35,RPL35A,RPL36,RPL36A,RPL37,RPL37A,RPL7A,RPL9,RPLP0,RPS10,RPS12,RPS13,RPS15,RPS15A,RPS16,RPS17,RPS18,RPS19,RPS2,RPS21,RPS23,RPS24,RPS25,RPS27A,RPS28,RPS29,RPS3,RPS3A,RPS4Y1,RPS6,RPS7,RPS8,RPSA
Regulation of eIF4 and p70S6K Signaling	27.6	0.175	ND	EIF3H,EIF4A3,FAU,PPP2CA,RAP1B,RPS10,RPS12,RPS13,RPS15,RPS15A,RPS16,RPS17,RPS18,RPS19,RPS2,RPS21,RPS23,RPS24,RPS25,RPS27A,RPS28,RPS29,RPS3,RPS3A,RPS4Y1,RPS6,RPS7,RPS8,RPSA
mTOR Signaling	27.2	0.148	1.342	EIF3H,EIF4A3,FAU,PPP2CA,RAP1B,RHOA,RHOQ,RPS10,RPS12,RPS13,RPS15,RPS15A,RPS16,RPS17,RPS18,RPS19,RPS2,RPS21,RPS23,RPS24,RPS25,RPS27A,RPS28,RPS29,RPS3,RPS3A,RPS4Y1,RPS6,RPS7,RPS8,RPSA
Mitochondrial Dysfunction	11	0.0936	ND	APP,ATP5F1A,COX4I1,COX5A,COX6A1,COX7C,CYCS,MAOB,MAPK8,MT-CO1,MT-CYB,MT-ND1,MT-ND2,MT-ND5,PRDX5,UCP2
Oxidative Phosphorylation	8.1	0.101	3.317	ATP5F1A,COX4I1,COX5A,COX6A1,COX7C,CYCS,MT-CO1,MT-CYB,MT-ND1,MT-ND2,MT-ND5
ILK Signaling	7.42	0.0684	2.111	ACTA1,ACTB,BMP2,FLNA,KRT18,MAPK8,MYH7,NACA,PPP2CA,RHOA,RHOQ,TMSB10/TMSB4X,VIM
Glycolysis I	6.86	0.231	2.449	ALDOA,ALDOB,ENO1,ENO3,GAPDH,TPI1
Caveolar-mediated Endocytosis Signaling	6.32	0.11	ND	ACTA1,ACTB,ALB,B2M,FLNA,FLOT1,FLOT2,SRC
Sumoylation Pathway	6.19	0.0874	0.378	MAPK8,NR3C1,PCNA,RAN,RHOA,RHOQ,SUMO1,TP53,UBE2I
Clathrin-mediated Endocytosis Signaling	5.58	0.057	ND	ACTA1,ACTB,ALB,APOA1,ARRB2,HSPA8,LYZ,RBP4,RPS27A,SRC,TF
Gluconeogenesis I	5.37	0.192	2.236	ALDOA,ALDOB,ENO1,ENO3,GAPDH

Sirtuin Signaling Pathway	5.31	0.0447	-0.333	APP,ATP5F1A,GLUD1,H1-0,LDHB,MT-CYB,MT-ND1,MT-ND2,MT-ND5,SOD1,TP53,TUBA1A,UCP2
Aryl Hydrocarbon Receptor Signaling	5.01	0.0629	1	ALDH7A1,CTSD,CYP1A1,CYP1A2,GSTA1,HSPB1,MAPK8,SRC,TP53
FXR/RXR Activation	4.54	0.0635	ND	ALB,APOA1,C3,CYP19A1,KNG1,MAPK8,RBP4,TF
HIF1α Signaling	4.53	0.0488	0.333	HSPA1A/HSPA1B,HSPA8,LDHB,RACK1,RAN,RAP1B,RPS6,TF,TP53,VIM
Superpathway of Melatonin Degradation	4.44	0.0923	2.449	CYP19A1,CYP1A1,CYP1A2,IL4I1,MAOB,POR
Germ Cell-Sertoli Cell Junction Signaling	4.39	0.0526	ND	ACTA1,ACTB,MAPK8,RAP1B,RHOA,RHOQ,SRC,TUBA1A,TUBB
Glucocorticoid Receptor Signaling	4.39	0.0325	ND	ACTB,ATP5F1A,GTF2A2,HSPA1A/HSPA1B,HSPA8,KRT13,KRT18,MAPK8,MT-CYB,NPPA,NR3C1,RAP1B,SRC,SUMO1,UBE2I
Acute Phase Response Signaling	4.22	0.05	1.342	ALB,APOA1,C3,FTL,MAPK8,NR3C1,RAP1B,RBP4,TF
Sucrose Degradation V (Mammalian)	4.16	0.333	ND	ALDOA,ALDOB,TPI1
Bupropion Degradation	4.06	0.16	2	CYP19A1,CYP1A1,CYP1A2,POR
NRF2-mediated Oxidative Stress Response	4.05	0.0476	2.236	ACTA1,ACTB,FTH1,FTL,GSTA1,MAPK8,RAP1B,SOD1,TXN
Production of Nitric Oxide and Reactive Oxygen Species in Macrophages	4.05	0.0476	2.121	ALB,APOA1,LYZ,MAPK8,PPP2CA,RAP1B,RBP4,RHOA,RHOQ
Xenobiotic Metabolism Signaling	3.98	0.0383	ND	ALDH7A1,CYP1A1,CYP1A2,FTL,GSTA1,IL4I1,MAOB,MAPK8,PPP2CA,RAP1B,SUMO1
Epithelial Adherens Junction Signaling	3.96	0.0526	ND	ACTA1,ACTB,MYH7,RAP1B,RHOA,SRC,TUBA1A,TUBB
Chemokine Signaling	3.93	0.075	2.449	CALM1 (includes others),GNAI1,MAPK8,RAP1B,RHOA,SRC

Gap Junction Signaling	3.9	0.0455	ND	ACTA1,ACTB,GJB2,GNAI1,NPR2,RAP1B,SRC,TUBA1A,TUBB
LXR/RXR Activation	3.78	0.0579	1.89	ALB,APOA1,C3,KNG1,LYZ,RBP4,TF
Acetone Degradation I (to Methylglyoxal)	3.68	0.129	2	CYP19A1,CYP1A1,CYP1A2,POR
14-3-3-mediated Signaling	3.65	0.0551	2	MAPK8,RAP1B,SRC,TUBA1A,TUBB,VIM,YWHAB
Maturity Onset Diabetes of Young (MODY) Signaling	3.57	0.0833	ND	ALDOB,APOA1,FABP1,GAPDH,UCP2
Tetrahydrobiopterin Biosynthesis I	3.57	0.667	ND	GCH1,PTS
Tetrahydrobiopterin Biosynthesis II	3.57	0.667	ND	GCH1,PTS
Protein Ubiquitination Pathway	3.51	0.0366	ND	B2M,HSPA1A/HSPA1B,HSPA8,HSPB1,PSMA2,PSMA7,PSMB3,PSMB5,RPS27A,UBE2I
Noradrenaline and Adrenaline Degradation	3.47	0.114	2	ADH5,ALDH7A1,IL4I1,MAOB
Iron homeostasis signaling pathway	3.45	0.0511	ND	BMP2,CIAO1,FTH1,FTL,HBB,LYRM4,TF
Virus Entry via Endocytic Pathways	3.35	0.0588	ND	ACTA1,ACTB,B2M,FLNA,RAP1B,SRC
Remodeling of Epithelial Adherens Junctions	3.31	0.0735	ND	ACTA1,ACTB,SRC,TUBA1A,TUBB
Melatonin Degradation II	3.27	0.5	ND	IL4I1,MAOB
Agrin Interactions at Neuromuscular Junction	3.26	0.0714	2.236	ACTA1,ACTB,MAPK8,RAP1B,SRC
Estrogen Biosynthesis	3.21	0.0976	2	CYP19A1,CYP1A1,CYP1A2,POR

IL-8 Signaling	3.16	0.04	2.828	ARRB2,CSTB,GNAI1,MAPK8,RAP1B,RHOA,RHOQ,SRC
GADD45 Signaling	3.13	0.158	ND	CCNB1,PCNA,TP53
Serotonin Receptor Signaling	3.13	0.093	ND	GCH1,IL4I1,MAOB,PTS
Dopamine Receptor Signaling	3.07	0.0649	ND	GCH1,IL4I1,MAOB,PPP2CA,PTS
Cardiomyocyte Differentiation via BMP Receptors	3.06	0.15	ND	BMP2,MYH7,NPPA
Role of PKR in Interferon Induction and Antiviral Response	3.02	0.0508	0	CYCS,HSPA1A/HSPA1B,HSPA8,MAPK8,TARBP2,TP53
Putrescine Degradation III	3	0.143	ND	ALDH7A1,IL4I1,MAOB
Spliceosomal Cycle	2.95	0.0833	2	EIF4A3,HSPA8,MAGOH,RBM8A
Reelin Signaling in Neurons	2.94	0.0492	2.449	APP,MAPK8,PAFAH1B1,RAP1B,RHOA,SRC
Xenobiotic Metabolism AHR Signaling Pathway	2.87	0.0588	1.342	ALDH7A1,CYP1A1,CYP1A2,GSTA1,SUMO1
Tec Kinase Signaling	2.85	0.0405	2.449	ACTA1,ACTB,GNAI1,MAPK8,RHOA,RHOQ,SRC
UVC-Induced MAPK Signaling	2.85	0.0784	2	MAPK8,RAP1B,SRC,TP53
p70S6K Signaling	2.82	0.0465	1.342	GNAI1,PPP2CA,RAP1B,RPS6,SRC,YWHAB
Ceramide Signaling	2.81	0.0568	2.236	CTSD,CYCS,MAPK8,PPP2CA,RAP1B
Actin Cytoskeleton Signaling	2.8	0.0352	2.449	ACTA1,ACTB,FLNA,KNG1,MYH7,RAP1B,RHOA,TMSB10/TMSB4X
Tryptophan Degradation X (Mammalian, via Tryptamine)	2.77	0.12	ND	ALDH7A1,IL4I1,MAOB

Death Receptor Signaling	2.72	0.0543	1.342	ACTA1,ACTB,CYCS,HSPB1,MAPK8
Unfolded Protein Response	2.7	0.0714	2	HSPA1A/HSPA1B,HSPA8,MAPK8,SEL1L
Huntington's Disease Signaling	2.69	0.0338	-1	ATP5F1A,CTSD,CYCS,HSPA1A/HSPA1B,HSPA8,MAPK8,RPS27A,TP53
Nicotine Degradation III	2.67	0.0702	2	CYP19A1,CYP1A1,CYP1A2,POR
Xenobiotic Metabolism CAR Signaling Pathway	2.63	0.037	1.89	ALDH7A1,CYP1A1,CYP1A2,GSTA1,PPP2CA,RACK1,SRC
Melatonin Degradation I	2.59	0.0667	2	CYP19A1,CYP1A1,CYP1A2,POR
Leukocyte Extravasation Signaling	2.58	0.0363	2.646	ACTA1,ACTB,GNAI1,MAPK8,RAP1B,RHOA,SRC
Sertoli Cell-Sertoli Cell Junction Signaling	2.57	0.0361	ND	ACTA1,ACTB,MAPK8,RAP1B,SRC,TUBA1A,TUBB
Dopamine Degradation	2.54	0.1	ND	ALDH7A1,IL4I1,MAOB
Factors Promoting Cardiogenesis in Vertebrates	2.49	0.04	2.449	BMP2,MAPK8,MYH7,NPPA,RHOA,SCN5A
Nicotine Degradation II	2.46	0.0615	2	CYP19A1,CYP1A1,CYP1A2,POR
Calcium Signaling	2.43	0.034	2	ACTA1,ATP2A1,ATP2B2,CALM1 (includes others),MYH7,RAP1B,TPM1
Paxillin Signaling	2.42	0.0463	2.236	ACTA1,ACTB,MAPK8,RAP1B,SRC
Serotonin Degradation	2.41	0.0597	2	ADH5,ALDH7A1,IL4I1,MAOB
Calcium Transport I	2.41	0.2	ND	ATP2A1,ATP2B2
Estrogen Receptor Signaling	2.35	0.0274	1	ATP5F1A,GNAI1,MT-CYB,NR3C1,PCNA,RAP1B,RHOA,SRC,TP53
Integrin Signaling	2.35	0.0329	2.646	ACTA1,ACTB,MAPK8,RAP1B,RHOA,RHOQ,SRC

Endocannabinoid Developing Neuron Pathway	2.3	0.0435	2.236	GNAI1,MAPK8,RAP1B,RHOA,SRC
CXCR4 Signaling	2.26	0.0359	2.449	GNAI1,MAPK8,RAP1B,RHOA,RHOQ,SRC
BER pathway	2.25	0.167	ND	FEN1,PCNA
Cholecystokinin/Gastrin-mediated Signaling	2.24	0.042	2.236	MAPK8,RAP1B,RHOA,RHOQ,SRC
Phenylalanine Degradation IV (Mammalian, via Side Chain)	2.12	0.143	ND	IL4I1,MAOB
Apelin Adipocyte Signaling Pathway	2.1	0.0488	2	GNAI1,GSTA1,MAPK8,SOD1
BAG2 Signaling Pathway	2.1	0.0698	ND	HSPA1A/HSPA1B,HSPA8,TP53
Cardiac Hypertrophy Signaling	2.07	0.0292	1.89	CALM1 (includes others),GNAI1,HSPB1,MAPK8,RAP1B,RHOA,RHOQ
Cellular Effects of Sildenafil (Viagra)	2.07	0.0382	ND	ACTA1,ACTB,CALM1 (includes others),MYH7,NPPA
IL-12 Signaling and Production in Macrophages	2.04	0.0376	ND	ALB,APOA1,LYZ,MAPK8,RBP4
MSP-RON Signaling In Cancer Cells Pathway	2.03	0.0373	2.236	FLNA,RAP1B,SRC,VIM,YWHAB
FAT10 Cancer Signaling Pathway	2.02	0.0652	ND	EEF1A1,PCNA,TP53
RhoGDI Signaling	2.01	0.0317	-2.236	ACTA1,ACTB,GNAI1,RHOA,RHOQ,SRC
Mismatch Repair in Eukaryotes	2	0.125	ND	FEN1,PCNA
Parkinson's Signaling	2	0.125	ND	CYCS,MAPK8
Hepatic Fibrosis Signaling Pathway	1.98	0.0239	3	CALM1 (includes others),FTH1,FTL,GNAI1,MAPK8,RAP1B,RHOA,RHOQ,TF

RAR Activation	1.96	0.0309	ND	ACTB,BMP2,MAPK8,RBP4,RPL7A,SRC
Signaling by Rho Family GTPases	1.95	0.0277	2.449	ACTA1,ACTB,GNAI1,MAPK8,RHOA,RHOQ,VIM
RAN Signaling	1.95	0.118	ND	CSE1L,RAN
Hereditary Breast Cancer Signaling	1.95	0.0357	ND	ACTB,CCNB1,RAP1B,RPS27A,TP53
Cell Cycle: G2/M DNA Damage Checkpoint Regulation	1.94	0.0612	ND	CCNB1,TP53,YWHAB
DNA damage-induced 14-3-3σ Signaling	1.86	0.105	ND	CCNB1,TP53
ATM Signaling	1.85	0.0412	0	CCNB1,MAPK8,PPP2CA,TP53
UVA-Induced MAPK Signaling	1.84	0.0408	ND	CYCS,MAPK8,RAP1B,TP53
VEGF Signaling	1.82	0.0404	2	ACTA1,ACTB,RAP1B,SRC
Molecular Mechanisms of Cancer	1.82	0.0225	ND	BMP2,CYCS,GNAI1,MAPK8,RAP1B,RHOA,RHOQ,SRC,TP53
Phagosome Maturation	1.82	0.0331	ND	B2M,CTSD,PRDX5,TUBA1A,TUBB
Apoptosis Signaling	1.81	0.04	1	CYCS,MAPK8,RAP1B,TP53
Regulation of Actin-based Motility by Rho	1.76	0.0388	2	ACTA1,ACTB,RHOA,RHOQ
Role of CHK Proteins in Cell Cycle Checkpoint Control	1.76	0.0526	ND	PCNA,PPP2CA,TP53
FAK Signaling	1.75	0.0385	ND	ACTA1,ACTB,RAP1B,SRC
Choline Degradation I	1.72	0.5	ND	ALDH7A1
S-methyl-5-thio-α-D-ribose 1-phosphate Degradation	1.72	0.5	ND	APIP

L-glutamine Biosynthesis II (tRNA-dependent)	1.72	0.5	ND	GATB
Formaldehyde Oxidation II (Glutathione-dependent)	1.72	0.5	ND	ADH5
Glutamate Biosynthesis II	1.72	0.5	ND	GLUD1
Glutamate Degradation X	1.72	0.5	ND	GLUD1
Induction of Apoptosis by HIV1	1.69	0.0492	ND	CYCS,MAPK8,TP53
LPS/IL-1 Mediated Inhibition of RXR Function	1.67	0.0267	ND	ALDH7A1,FABP1,GSTA1,IL4I1,MAOB,MAPK8
Glioblastoma Multiforme Signaling	1.67	0.0303	1.342	RAP1B,RHOA,RHOQ,SRC,TP53
Cardiac Hypertrophy Signaling (Enhanced)	1.67	0.0202	3.162	ATP2A1,CALM1 (includes others),FGFR2,GNAI1,HSPB1,MAPK8,NPPA,RAP1B,RHOA,RPS6
Vitamin-C Transport	1.66	0.0833	ND	GJB2,TXN
Tight Junction Signaling	1.64	0.0298	ND	ACTA1,ACTB,MYH7,PPP2CA,RHOA
NGF Signaling	1.62	0.0351	2	MAPK8,RAP1B,RHOA,TP53
PXR/RXR Activation	1.61	0.0462	ND	CYP1A2,GSTA1,NR3C1
Apelin Endothelial Signaling Pathway	1.61	0.0348	2	CALM1 (includes others),GNAI1,MAPK8,RAP1B
Role of Tissue Factor in Cancer	1.6	0.0345	ND	ARRB2,RAP1B,SRC,TP53
GNRH Signaling	1.59	0.0289	2	CALM1 (includes others),GNAI1,MAPK8,RAP1B,SRC
Nur77 Signaling in T Lymphocytes	1.56	0.0441	ND	CALM1 (includes others),CYCS,PCNA
NADH Repair	1.55	0.333	ND	GAPDH

1,25-dihydroxyvitamin D3 Biosynthesis	1.55	0.333	ND	POR
Thyroid Hormone Biosynthesis	1.55	0.333	ND	CTSD
Sonic Hedgehog Signaling	1.51	0.069	ND	ARRB2,CCNB1
CCR3 Signaling in Eosinophils	1.51	0.0323	ND	CALM1 (includes others),GNAI1,RAP1B,RHOA
ERK5 Signaling	1.5	0.0417	ND	RAP1B,SRC,YWHAB
PI3K/AKT Signaling	1.49	0.027	1.342	CDC37,PPP2CA,RAP1B,TP53,YWHAB
Glioma Invasiveness Signaling	1.48	0.0411	ND	RAP1B,RHOA,RHOQ
IL-6 Signaling	1.48	0.0317	2	CYP19A1,HSPB1,MAPK8,RAP1B
Atherosclerosis Signaling	1.47	0.0315	ND	ALB,APOA1,LYZ,RBP4
Estrogen-Dependent Breast Cancer Signaling	1.47	0.0405	ND	CYP19A1,RAP1B,SRC
Hypoxia Signaling in the Cardiovascular System	1.47	0.0405	ND	SUMO1,TP53,UBE2I
Regulation Of The Epithelial Mesenchymal Transition By Growth Factors Pathway	1.46	0.0266	1.342	FGFR2,MAPK8,RAP1B,RHOA,VIM
Colorectal Cancer Metastasis Signaling	1.46	0.0237	1.633	MAPK8,RAP1B,RHOA,RHOQ,SRC,TP53
Toll-like Receptor Signaling	1.44	0.0395	ND	MAPK8,RPS27A,TOLLIP
Macropinocytosis Signaling	1.44	0.0395	ND	RAP1B,RHOA,SRC
Gα12/13 Signaling	1.43	0.0305	2	MAPK8,RAP1B,RHOA,SRC

Ethanol Degradation II	1.43	0.0625	ND	ADH5,ALDH7A1
Antiproliferative Role of Somatostatin Receptor 2	1.43	0.039	ND	NPR2,RAP1B,SRC
STAT3 Pathway	1.39	0.0296	2	FGFR2,MAPK8,RAP1B,SRC
Adrenomedullin signaling pathway	1.39	0.0254	1.342	C3,CALM1 (includes others),MAPK8,NPR2,RAP1B
Cytotoxic T Lymphocyte-mediated Apoptosis of Target Cells	1.38	0.0588	ND	B2M,CYCS
Inhibition of Angiogenesis by TSP1	1.38	0.0588	ND	MAPK8,TP53
Cyclins and Cell Cycle Regulation	1.37	0.037	ND	CCNB1,PPP2CA,TP53
Actin Nucleation by ARP-WASP Complex	1.37	0.037	ND	RAP1B,RHOA,RHOQ
PEDF Signaling	1.36	0.0366	ND	RAP1B,RHOA,TP53
Ovarian Cancer Signaling	1.35	0.0288	ND	CGA,RAP1B,SRC,TP53
ERK/MAPK Signaling	1.35	0.0248	1	HSPB1,PPP2CA,RAP1B,SRC,YWHAB
TR/RXR Activation	1.33	0.0357	ND	ATP2A1,ENO1,UCP2
Creatine-phosphate Biosynthesis	1.33	0.2	ND	CKB
CMP-N-acetylneuraminate Biosynthesis I (Eukaryotes)	1.33	0.2	ND	CMAS
Lysine Degradation II	1.33	0.2	ND	ALDH7A1
Lysine Degradation V	1.33	0.2	ND	ALDH7A1

BMP signaling pathway	1.32	0.0353	ND	BMP2,MAPK8,RAP1B
Endocannabinoid Cancer Inhibition Pathway	1.31	0.028	-1	GNAI1,RHOA,SRC,VIM
Xenobiotic Metabolism General Signaling Pathway	1.31	0.028	2	FTL,GSTA1,MAPK8,RAP1B
Complement System	1.31	0.0541	ND	C3,C8B
PDGF Signaling	1.31	0.0349	ND	MAPK8,RAP1B,SRC
Thrombin Signaling	1.3	0.024	2.236	GNAI1,RAP1B,RHOA,RHOQ,SRC
Corticotropin Releasing Hormone Signaling	1.3	0.0276	ND	CALM1 (includes others),GNAI1,NPR2,RAP1B
Docosahexaenoic Acid (DHA) Signaling	1.29	0.0526	ND	APP,CYCS
Antigen Presentation Pathway	1.27	0.0513	ND	B2M,PSMB5
RANK Signaling in Osteoclasts	1.27	0.0337	ND	CALM1 (includes others),MAPK8,SRC
Role of NFAT in Cardiac Hypertrophy	1.26	0.0234	1.342	CALM1 (includes others),GNAI1,MAPK8,RAP1B,SRC
Regulation of IL-2 Expression in Activated and Anergic T Lymphocytes	1.26	0.0333	ND	CALM1 (includes others),MAPK8,RAP1B
Arginine Biosynthesis IV	1.25	0.167	ND	GLUD1
Pyruvate Fermentation to Lactate	1.25	0.167	ND	LDHB

Tryptophan Degradation to 2-amino-3-carboxymuconate Semialdehyde	1.25	0.167	ND	AFMID
IL-1 Signaling	1.24	0.0326	ND	GNAI1,MAPK8,TOLLIP
Mechanisms of Viral Exit from Host Cells	1.23	0.0488	ND	ACTA1,ACTB
Role of Osteoblasts, Osteoclasts and Chondrocytes in Rheumatoid Arthritis	1.23	0.0229	ND	ALPL,BMP2,CALM1 (includes others),MAPK8,SRC
MIF Regulation of Innate Immunity	1.22	0.0476	ND	MAPK8,TP53
Fcγ Receptor-mediated Phagocytosis in Macrophages and Monocytes	1.21	0.0319	ND	ACTA1,ACTB,SRC
CCR5 Signaling in Macrophages	1.21	0.0319	ND	CALM1 (includes others),GNAI1,MAPK8
α-Adrenergic Signaling	1.19	0.0312	ND	CALM1 (includes others),GNAI1,RAP1B
TGF-β Signaling	1.19	0.0312	ND	BMP2,MAPK8,RAP1B
Thioredoxin Pathway	1.19	0.143	ND	TXN
Amyotrophic Lateral Sclerosis Signaling	1.18	0.0309	ND	CYCS,SOD1,TP53
eNOS Signaling	1.18	0.0252	0	CALM1 (includes others),HSPA1A/HSPA1B,HSPA8,KNG1
p53 Signaling	1.17	0.0306	ND	MAPK8,PCNA,TP53
Coronavirus Replication Pathway	1.16	0.0444	ND	TUBA1A,TUBB

Nitric Oxide Signaling in the Cardiovascular System	1.16	0.0303	ND	ATP2A1,CALM1 (includes others),KNG1
Apelin Cardiomyocyte Signaling Pathway	1.16	0.0303	ND	ATP2A1,GNAI1,MAPK8
Dopamine-DARPP32 Feedback in cAMP Signaling	1.15	0.0245	ND	ATP2A1,CALM1 (includes others),GNAI1,PPP2CA
HMGB1 Signaling	1.13	0.0242	2	MAPK8,RAP1B,RHOA,RHOQ
Superoxide Radicals Degradation	1.13	0.125	ND	SOD1
SAPK/JNK Signaling	1.13	0.0294	ND	MAPK8,RAP1B,TP53
IGF-1 Signaling	1.11	0.0288	ND	MAPK8,RAP1B,YWHAB
Neuregulin Signaling	1.1	0.0286	ND	RAP1B,RPS6,SRC
Synaptogenesis Signaling Pathway	1.1	0.0192	1.633	CALM1 (includes others),HSPA8,PAFAH1B1,RAP1B,RHOA,SRC
T Cell Receptor Signaling	1.09	0.0283	ND	CALM1 (includes others),MAPK8,RAP1B
Melanoma Signaling	1.08	0.04	ND	RAP1B,TP53
Myc Mediated Apoptosis Signaling	1.08	0.04	ND	CYCS,TP53
TNFR1 Signaling	1.08	0.04	ND	CYCS,MAPK8
Telomerase Signaling	1.08	0.028	ND	PPP2CA,RAP1B,TP53
Erythropoietin Signaling	1.07	0.0231	1	HBB,RAP1B,SRC,TP53
Wnt/β-catenin Signaling	1.07	0.0231	ND	PPP2CA,RPS27A,SRC,TP53
CDK5 Signaling	1.07	0.0278	ND	MAPK8,PPP2CA,RAP1B
Insulin Secretion Signaling Pathway	1.07	0.0205	2.236	EIF4A3,RAP1B,SEC61A2,SRC,SSR1
UVB-Induced MAPK Signaling	1.05	0.0385	ND	MAPK8,TP53
Glioma Signaling	1.05	0.0273	ND	CALM1 (includes others),RAP1B,TP53

Opioid Signaling Pathway	1.05	0.0202	2	ARRB2,CALM1 (includes others),GNAI1,RAP1B,SRC
Protein Kinase A Signaling	1.05	0.0175	1.633	CALM1 (includes others),FLNA,GNAI1,H1-0,RAP1B,RHOA,YWHAB
CD27 Signaling in Lymphocytes	1.04	0.0377	ND	CYCS,MAPK8
NF-κB Signaling	1.03	0.0223	2	BMP2,FGFR2,MAPK8,RAP1B
EGF Signaling	1.01	0.0364	ND	MAPK8,SRC
fMLP Signaling in Neutrophils	1	0.0259	ND	CALM1 (includes others),GNAI1,RAP1B
Neuroprotective Role of THOP1 in Alzheimer's Disease	1	0.0259	ND	APP,KNG1,PRSS2
Purine Nucleotides De Novo Biosynthesis II	1	0.0909	ND	ADSS1
Sphingosine-1-phosphate Signaling	0.991	0.0256	ND	GNAI1,RHOA,RHOQ
Systemic Lupus Erythematosus In T Cell Signaling Pathway	0.991	0.018	1.633	B2M,GNAI1,PPP2CA,RAP1B,RHOA,RHOQ
Endothelin-1 Signaling	0.975	0.0213	2	GNAI1,MAPK8,RAP1B,SRC
Role of NANOG in Mammalian Embryonic Stem Cell Pluripotency	0.975	0.0252	ND	BMP2,RAP1B,TP53
MSP-RON Signaling Pathway	0.975	0.0345	ND	ACTA1,ACTB
Cancer Drug Resistance By Drug Efflux	0.975	0.0345	ND	RAP1B,TP53

Synaptic Long Term Depression	0.967	0.0212	1	GNAI1,NPR2,PPP2CA,RAP1B
Ephrin Receptor Signaling	0.967	0.0212	2	GNAI1,RAP1B,RHOA,SRC
NAD Biosynthesis II (from tryptophan)	0.963	0.0833	ND	AFMID
Mineralocorticoid Biosynthesis	0.963	0.0833	ND	GSTA1
Rac Signaling	0.959	0.0248	ND	MAPK8,RAP1B,RHOA
Semaphorin Signaling in Neurons	0.951	0.0333	ND	RHOA,RHOQ
Endometrial Cancer Signaling	0.951	0.0333	ND	RAP1B,TP53
G Beta Gamma Signaling	0.951	0.0246	ND	GNAI1,RAP1B,SRC
Phospholipase C Signaling	0.951	0.0188	2.236	CALM1 (includes others),RAP1B,RHOA,RHOQ,SRC
Agranulocyte Adhesion and Diapedesis	0.951	0.0208	ND	ACTA1,ACTB,GNAI1,MYH7
PCP pathway	0.951	0.0333	ND	MAPK8,RHOA
Xenobiotic Metabolism PXR Signaling Pathway	0.951	0.0208	2	ALDH7A1,GSTA1,IL4I1,MAOB
RhoA Signaling	0.943	0.0244	ND	ACTA1,ACTB,RHOA
IL-2 Signaling	0.939	0.0328	ND	MAPK8,RAP1B
Glucocorticoid Biosynthesis	0.932	0.0769	ND	GSTA1
Gαi Signaling	0.928	0.024	ND	GNAI1,RAP1B,SRC
Natural Killer Cell Signaling	0.921	0.0203	1	B2M,HSPA1A/HSPA1B,HSPA8,RAP1B
Senescence Pathway	0.907	0.0182	1.342	CALM1 (includes others),CCNB1,PPP2CA,RAP1B,TP53

White Adipose Tissue Browning Pathway	0.9	0.0233	ND	FGFR2,LDHB,NPPA
Calcium-induced T Lymphocyte Apoptosis	0.883	0.0303	ND	ATP2A1,CALM1 (includes others)
Mitotic Roles of Polo-Like Kinase	0.883	0.0303	ND	CCNB1,PPP2CA
Cell Cycle: G1/S Checkpoint Regulation	0.873	0.0299	ND	RPL11,TP53
Adipogenesis pathway	0.863	0.0224	ND	BMP2,FGFR2,TP53
Androgen Signaling	0.851	0.0221	ND	CALM1 (includes others),GNAI1,SRC
SPINK1 General Cancer Pathway	0.851	0.029	ND	PRSS2,RAP1B
Granzyme B Signaling	0.848	0.0625	ND	CYCS
Androgen Biosynthesis	0.848	0.0625	ND	GSTA1
GM-CSF Signaling	0.842	0.0286	ND	RACK1,RAP1B
PI3K Signaling in B Lymphocytes	0.836	0.0217	ND	C3,CALM1 (includes others),RAP1B
Small Cell Lung Cancer Signaling	0.833	0.0282	ND	CYCS,TP53
Insulin Receptor Signaling	0.824	0.0214	ND	MAPK8,RAP1B,RHOQ
Melatonin Signaling	0.821	0.0278	ND	CALM1 (includes others),GNAI1
Basal Cell Carcinoma Signaling	0.821	0.0278	ND	BMP2,TP53
Ephrin B Signaling	0.821	0.0278	ND	GNAI1,RHOA
Histamine Degradation	0.821	0.0588	ND	ALDH7A1

Non-Small Cell Lung Cancer Signaling	0.812	0.0274	ND	RAP1B,TP53
Regulation of Cellular Mechanics by Calpain Protease	0.804	0.027	ND	RAP1B,SRC
FcγRIIB Signaling in B Lymphocytes	0.793	0.0267	ND	MAPK8,RAP1B
GDNF Family Ligand-Receptor Interactions	0.785	0.0263	ND	MAPK8,RAP1B
Neurotrophin/TRK Signaling	0.785	0.0263	ND	MAPK8,RAP1B
Sperm Motility	0.783	0.0179	ND	CALM1 (includes others),FGFR2,NPPA,SRC
Granzyme A Signaling	0.777	0.0526	ND	H1-0
Oxidative Ethanol Degradation III	0.777	0.0526	ND	ALDH7A1
Relaxin Signaling	0.762	0.02	ND	GNAI1,NPR2,RAP1B
Thyroid Cancer Signaling	0.759	0.0253	ND	RAP1B,TP53
BEX2 Signaling Pathway	0.759	0.0253	ND	MAPK8,PPP2CA
Fatty Acid α-Oxidation	0.757	0.05	ND	ALDH7A1
Role of BRCA1 in DNA Damage Response	0.75	0.025	ND	ACTB,TP53
IL-17 Signaling	0.75	0.025	ND	MAPK8,RAP1B
Renal Cell Carcinoma Signaling	0.75	0.025	ND	RAP1B,RPS27A
Role of MAPK Signaling in the Pathogenesis of Influenza	0.75	0.025	ND	MAPK8,RAP1B
Prolactin Signaling	0.742	0.0247	ND	NR3C1,RAP1B

Role of Macrophages, Fibroblasts and Endothelial Cells in Rheumatoid Arthritis	0.742	0.016	ND	CALM1 (includes others),PRSS2,RAP1B,RHOA,SRC
Endoplasmic Reticulum Stress Pathway	0.738	0.0476	ND	MAPK8
LPS-stimulated MAPK Signaling	0.726	0.0241	ND	MAPK8,RAP1B
Pyrimidine Deoxyribonucleotides De Novo Biosynthesis I	0.721	0.0455	ND	NME2
HER-2 Signaling in Breast Cancer	0.719	0.0238	ND	RAP1B,TP53
FGF Signaling	0.719	0.0238	ND	FGFR2,MAPK8
Aldosterone Signaling in Epithelial Cells	0.717	0.019	ND	HSPA1A/HSPA1B,HSPA8,HSPB1
Gαq Signaling	0.717	0.019	ND	CALM1 (includes others),RHOA,RHOQ
HIPPO signaling	0.71	0.0235	ND	PPP2CA,YWHAB
IL-4 Signaling	0.71	0.0235	ND	NR3C1,RAP1B
Tryptophan Degradation III (Eukaryotic)	0.703	0.0435	ND	AFMID
Ethanol Degradation IV	0.703	0.0435	ND	ALDH7A1
IL-22 Signaling	0.686	0.0417	ND	MAPK8
Tumoricidal Function of Hepatic Natural Killer Cells	0.686	0.0417	ND	CYCS
Role of JAK1, JAK2 and TYK2 in Interferon Signaling	0.686	0.0417	ND	CGA

Glutathione Redox Reactions I	0.686	0.0417	ND	GSTA1
TCA Cycle II (Eukaryotic)	0.686	0.0417	ND	CS
CTLA4 Signaling in Cytotoxic T Lymphocytes	0.68	0.0225	ND	B2M,PPP2CA
Crosstalk between Dendritic Cells and Natural Killer Cells	0.68	0.0225	ND	ACTA1,ACTB
OX40 Signaling Pathway	0.674	0.0222	ND	B2M,MAPK8
IL-17A Signaling in Gastric Cells	0.672	0.04	ND	MAPK8
Role of JAK Family Kinases in IL-6-type Cytokine Signaling	0.672	0.04	ND	MAPK8
Prostate Cancer Signaling	0.666	0.022	ND	RAP1B,TP53
Lipid Antigen Presentation by CD1	0.656	0.0385	ND	B2M
Apelin Liver Signaling Pathway	0.656	0.0385	ND	MAPK8
Melanocyte Development and Pigmentation Signaling	0.646	0.0213	ND	RAP1B,SRC
ErbB Signaling	0.646	0.0213	ND	MAPK8,RAP1B
T Cell Exhaustion Signaling Pathway	0.631	0.0171	ND	MAPK8,PPP2CA,RAP1B
Bladder Cancer Signaling	0.625	0.0206	ND	RAP1B,TP53
Cdc42 Signaling	0.625	0.017	ND	B2M,MAPK8,SRC

Salvage Pathways of Pyrimidine Ribonucleotides	0.62	0.0204	ND	MAPK8,NME2
Role of p14/p19ARF in Tumor Suppression	0.614	0.0345	ND	TP53
Role of NFAT in Regulation of the Immune Response	0.602	0.0166	ND	CALM1 (includes others),GNAI1,RAP1B
TNFR2 Signaling	0.602	0.0333	ND	MAPK8
Kinetochore Metaphase Signaling Pathway	0.6	0.0198	ND	CCNB1,H2AZ1
Chronic Myeloid Leukemia Signaling	0.588	0.0194	ND	RAP1B,TP53
Mouse Embryonic Stem Cell Pluripotency	0.588	0.0194	ND	RAP1B,TP53
NER Pathway	0.588	0.0194	ND	PCNA,UBE2I
B Cell Receptor Signaling	0.582	0.0161	ND	CALM1 (includes others),MAPK8,RAP1B
4-1BB Signaling in T Lymphocytes	0.578	0.0312	ND	MAPK8
Glutathione-mediated Detoxification	0.578	0.0312	ND	GSTA1
PAK Signaling	0.57	0.0189	ND	MAPK8,RAP1B
PPARα/RXRα Activation	0.561	0.0157	ND	APOA1,MAPK8,RAP1B
MIF-mediated Glucocorticoid Regulation	0.556	0.0294	ND	NR3C1
Retinoate Biosynthesis I	0.556	0.0294	ND	BMP2

Regulation of the Epithelial-Mesenchymal Transition Pathway	0.556	0.0156	ND	FGFR2,RAP1B,RHOA
Pancreatic Adenocarcinoma Signaling	0.554	0.0183	ND	MAPK8,TP53
Antioxidant Action of Vitamin C	0.554	0.0183	ND	MAPK8,TXN
Coagulation System	0.545	0.0286	ND	KNG1
TWEAK Signaling	0.545	0.0286	ND	CYCS
Type I Diabetes Mellitus Signaling	0.544	0.018	ND	CYCS,MAPK8
Cell Cycle Regulation by BTG Family Proteins	0.524	0.027	ND	PPP2CA
Fc Epsilon RI Signaling	0.511	0.0171	ND	MAPK8,RAP1B
Renin-Angiotensin Signaling	0.507	0.0169	ND	MAPK8,RAP1B
p38 MAPK Signaling	0.507	0.0169	ND	HSPB1,TP53
GP6 Signaling Pathway	0.502	0.0168	ND	CALM1 (includes others),RAP1B
HGF Signaling	0.498	0.0167	ND	MAPK8,RAP1B
April Mediated Signaling	0.496	0.025	ND	MAPK8
IL-15 Production	0.492	0.0165	ND	FGFR2,SRC
CD28 Signaling in T Helper Cells	0.492	0.0165	ND	CALM1 (includes others),MAPK8
B Cell Activating Factor Signaling	0.488	0.0244	ND	MAPK8
nNOS Signaling in Skeletal Muscle Cells	0.488	0.0244	ND	CALM1 (includes others)

Inhibition of ARE-Mediated mRNA Degradation Pathway	0.488	0.0164	ND	PPP2CA,YWHAB
Intrinsic Prothrombin Activation Pathway	0.479	0.0238	ND	KNG1
Pyrimidine Ribonucleotides Interconversion	0.479	0.0238	ND	NME2
Axonal Guidance Signaling	0.476	0.0121	ND	BMP2,GNAI1,RAP1B,RHOA,TUBA1A,TUBB
AMPK Signaling	0.475	0.014	ND	ACTB,AK2,PPP2CA
Breast Cancer Regulation by Stathmin1	0.475	0.0118	0.378	GPRC6A,PPP2CA,RAP1B,RHOA,TP53,TUBA1A,TUBB
Oncostatin M Signaling	0.471	0.0233	ND	RAP1B
P2Y Purigenic Receptor Signaling Pathway	0.466	0.0157	ND	GNAI1,RAP1B
Pyrimidine Ribonucleotides De Novo Biosynthesis	0.462	0.0227	ND	NME2
Apelin Pancreas Signaling Pathway	0.462	0.0227	ND	MAPK8
Endocannabinoid Neuronal Synapse Pathway	0.461	0.0156	ND	GNAI1,MAPK8
Synaptic Long Term Potentiation	0.457	0.0155	ND	CALM1 (includes others),RAP1B
iNOS Signaling	0.455	0.0222	ND	CALM1 (includes others)
Osteoarthritis Pathway	0.455	0.0136	ND	ALPL,BMP2,RBP4

Role of Oct4 in Mammalian Embryonic Stem Cell Pluripotency	0.447	0.0217	ND	TP53
PFKFB4 Signaling Pathway	0.447	0.0217	ND	TP53
nNOS Signaling in Neurons	0.44	0.0213	ND	CALM1 (includes others)
Ephrin A Signaling	0.44	0.0213	ND	RHOA
Phagosome Formation	0.44	0.015	ND	RHOA,RHOQ
Human Embryonic Stem Cell Pluripotency	0.432	0.0148	ND	BMP2,FGFR2
cAMP-mediated signaling	0.429	0.0132	ND	CALM1 (includes others),GNAI1,SRC
PTEN Signaling	0.428	0.0147	ND	FGFR2,RAP1B
Systemic Lupus Erythematosus Signaling	0.427	0.0131	ND	C8B,KNG1,RAP1B
Autoimmune Thyroid Disease Signaling	0.426	0.0204	ND	CGA
Assembly of RNA Polymerase II Complex	0.419	0.02	ND	GTF2A2
Amyloid Processing	0.419	0.02	ND	APP
Semaphorin Neuronal Repulsive Signaling Pathway	0.416	0.0144	ND	RHOA,TP53
Cardiac β-adrenergic Signaling	0.409	0.0142	ND	ATP2A1,PPP2CA

D-myo-inositol (1,4,5,6)-Tetrakisphosphate Biosynthesis	0.405	0.0141	ND	ALPL,ATP1A1
D-myo-inositol (3,4,5,6)-tetrakisphosphate Biosynthesis	0.405	0.0141	ND	ALPL,ATP1A1
Lymphotoxin β Receptor Signaling	0.399	0.0189	ND	CYCS
Phototransduction Pathway	0.399	0.0189	ND	OPN4
Role of IL-17A in Arthritis	0.393	0.0185	ND	MAPK8
Cell Cycle Control of Chromosomal Replication	0.381	0.0179	ND	PCNA
CNTF Signaling	0.375	0.0175	ND	RAP1B
Glutamate Receptor Signaling	0.375	0.0175	ND	CALM1 (includes others)
Role of Pattern Recognition Receptors in Recognition of Bacteria and Viruses	0.364	0.013	ND	C3,MAPK8
PKCθ Signaling in T Lymphocytes	0.36	0.0129	ND	MAPK8,RAP1B
Retinoic acid Mediated Apoptosis Signaling	0.359	0.0167	ND	CYCS
SPINK1 Pancreatic Cancer Pathway	0.359	0.0167	ND	PRSS2

3-phosphoinositide Degradation	0.357	0.0128	ND	ALPL,ATP1A1
D-myo-inositol-5-phosphate Metabolism	0.354	0.0127	ND	ALPL,ATP1A1
Autophagy	0.354	0.0164	ND	CTSD
Necroptosis Signaling Pathway	0.354	0.0127	ND	GLUD1,TP53
Activation of IRF by Cytosolic Pattern Recognition Receptors	0.343	0.0159	ND	MAPK8
Thrombopoietin Signaling	0.343	0.0159	ND	RAP1B
Role of PI3K/AKT Signaling in the Pathogenesis of Influenza	0.338	0.0156	ND	GNAI1
IL-17A Signaling in Airway Cells	0.338	0.0156	ND	MAPK8
CD40 Signaling	0.333	0.0154	ND	MAPK8
ErbB2-ErbB3 Signaling	0.333	0.0154	ND	RAP1B
Pyridoxal 5'-phosphate Salvage Pathway	0.328	0.0152	ND	MAPK8
3-phosphoinositide Biosynthesis	0.326	0.012	ND	ALPL,ATP1A1
ErbB4 Signaling	0.323	0.0149	ND	RAP1B
Role of JAK1 and JAK3 in γc Cytokine Signaling	0.314	0.0145	ND	RAP1B

G-Protein Coupled Receptor Signaling	0.314	0.011	ND	GNAI1,RAP1B,SRC
IL-10 Signaling	0.31	0.0143	ND	MAPK8
Systemic Lupus Erythematosus In B Cell Signaling Pathway	0.31	0.0109	ND	CALM1 (includes others),RAP1B,SRC
GPCR-Mediated Integration of Enteroendocrine Signaling Exemplified by an L Cell	0.298	0.0137	ND	GNAI1
IL-15 Signaling	0.289	0.0133	ND	RAP1B
Angiopoietin Signaling	0.289	0.0133	ND	RAP1B
NF-κB Activation by Viruses	0.286	0.0132	ND	RAP1B
Dendritic Cell Maturation	0.279	0.0109	ND	B2M,MAPK8
Hepatic Fibrosis / Hepatic Stellate Cell Activation	0.274	0.0108	ND	FGFR2,MYH7
IL-3 Signaling	0.274	0.0127	ND	RAP1B
FLT3 Signaling in Hematopoietic Progenitor Cells	0.271	0.0125	ND	RAP1B
JAK/Stat Signaling	0.271	0.0125	ND	RAP1B
Neuroinflammation Signaling Pathway	0.261	0.01	ND	APP,B2M,MAPK8
VEGF Family Ligand-Receptor Interactions	0.256	0.0119	ND	RAP1B
Allograft Rejection Signaling	0.25	0.0116	ND	B2M

Superpathway of Inositol Phosphate Compounds	0.245	0.0101	ND	ALPL,ATP1A1
Acute Myeloid Leukemia Signaling	0.24	0.0112	ND	RAP1B
GABA Receptor Signaling	0.223	0.0105	ND	RPS27A
Communication between Innate and Adaptive Immune Cells	0.22	0.0104	ND	B2M
Neuropathic Pain Signaling In Dorsal Horn Neurons	0.207	0.0099	ND	SRC
Hepatic Cholestasis	0	0.0054	ND	MAPK8
Airway Pathology in Chronic Obstructive Pulmonary Disease	0	0.0085	ND	RBP4
iCOS-iCOSL Signaling in T Helper Cells	0	0.009	ND	CALM1 (includes others)
CREB Signaling in Neurons	0	0.0084	1.342	CALM1 (includes others),FGFR2,GNAI1,GPRC6A,RAP1B
Type II Diabetes Mellitus Signaling	0	0.007	ND	MAPK8
Gαs Signaling	0	0.0094	ND	SRC
Granulocyte Adhesion and Diapedesis	0	0.0058	ND	GNAI1
PPAR Signaling	0	0.0095	ND	RAP1B
GPCR-Mediated Nutrient Sensing in Enteroendocrine Cells	0	0.0089	ND	GNAI1

PD-1, PD-L1 cancer immunotherapy pathway	0	0.0094	ND	B2M
HOTAIR Regulatory Pathway	0	0.0063	ND	VIM
Tumor Microenvironment Pathway	0	0.0057	ND	RAP1B
MSP-RON Signaling In Macrophage Pathway	0	0.0089	ND	RAP1B

Supplementary Table.3 IPA pathway analysis between PGC-1α and OPA1.

Pathways from PGC-1α to OPA1

PGC-1α ⇨ Symbol ⇨ OPA1

	Symbol	Entrez Gene Name	Location	Family	Entrez Gene ID for Human
1	BAX	BCL2 associated X, apoptosis regulator	Cytoplasm	transporter	581
2	BID	BH3 interacting domain death agonist	Cytoplasm	other	637
3	BNIP3	BCL2 interacting protein 3	Cytoplasm	other	664
4	CLUH	clustered mitochondria homolog	Cytoplasm	translation regulator	23277
5	COX4I1	cytochrome c oxidase subunit 4I1	Cytoplasm	enzyme	1327
6	COX5A	cytochrome c oxidase subunit 5A	Cytoplasm	enzyme	9377
7	EGFR	epidermal growth factor receptor	Plasma Membrane	kinase	1956
8	ESR1	estrogen receptor 1	Nucleus	ligand-dependent nuclear recepto	2099
9	ESR2	estrogen receptor 2	Nucleus	ligand-dependent nuclear recepto	2100
10	HNF4A	hepatocyte nuclear factor 4 alpha	Nucleus	transcription regulator	3172
11	MAPK14	mitogen-activated protein kinase 14	Cytoplasm	kinase	1432
12	MYC	MYC proto-oncogene, bHLH transcription factor	Nucleus	transcription regulator	4609
13	NR4A2	nuclear receptor subfamily 4 group A member 2	Nucleus	ligand-dependent nuclear recepto	4929
14	PDK4	pyruvate dehydrogenase kinase 4	Cytoplasm	kinase	5166
15	PPRC1	PPARG related coactivator 1	Nucleus	transcription regulator	23082
16	PRDX3	peroxiredoxin 3	Cytoplasm	enzyme	10935
17	SIRT3	sirtuin 3	Cytoplasm	enzyme	23410
18	SMAD3	SMAD family member 3	Nucleus	transcription regulator	4088
19	STAT3	signal transducer and activator of transcription 3	Nucleus	transcription regulator	6774
20	TP53	tumor protein p53	Nucleus	transcription regulator	7157
21	VIRMA	vir like m6A methyltransferase associated	Nucleus	other	25962

Pathways from OPA1 to PGC-1α

PGC-1α ⇨ Symbol ⇨ OPA1

	Symbol	Entrez Gene Name	Location	Family	Entrez Gene ID for Human
1	ADRB2	adrenoceptor beta 2	Plasma Membrane	G-protein coupled receptor	154
2	aldosterone		Other	chemical - endogenous mammalian	
3	APOA1	apolipoprotein A1	Extracellular Space	transporter	335
4	CDK9	cyclin dependent kinase 9	Nucleus	kinase	1025

5	cholesterol		Other	chemical - endogenous mammalian	
6	cholic acid		Other	chemical - endogenous mammalian	
7	CLUH	clustered mitochondria homolog	Cytoplasm	translation regulator	23277
8	D-glucose		Other	chemical - endogenous mammalian	
9	DDIT3	DNA damage inducible transcript 3	Nucleus	transcription regulator	1649
10	EGFR	epidermal growth factor receptor	Plasma Membrane	kinase	1956
11	ESR2	estrogen receptor 2	Nucleus	ligand-dependent nuclear recepto	2100
12	FGF21	fibroblast growth factor 21	Extracellular Space	growth factor	26291
13	HTT	huntingtin	Cytoplasm	transcription regulator	3064
14	IL1B	interleukin 1 beta	Extracellular Space	cytokine	3553
15	LIPE	lipase E, hormone sensitive type	Cytoplasm	enzyme	3991
16	MAPK11	mitogen-activated protein kinase 11	Cytoplasm	kinase	5600
17	MAPK14	mitogen-activated protein kinase 14	Cytoplasm	kinase	1432
18	NFkB (complex)		Nucleus	complex	
19	NR4A2	nuclear receptor subfamily 4 group A member 2	Nucleus	ligand-dependent nuclear recepto	4929
20	OMA1	OMA1 zinc metallopeptidase	Cytoplasm	peptidase	115209
22	PNPLA2	patatin like phospholipase domain containing 2	Cytoplasm	enzyme	57104
24	PTPN1	protein tyrosine phosphatase non-receptor type 1	Cytoplasm	phosphatase	5770
25	SIRT3	sirtuin 3	Cytoplasm	enzyme	23410
26	SMAD3	SMAD family member 3	Nucleus	transcription regulator	4088
27	STAT3	signal transducer and activator of transcription 3	Nucleus	transcription regulator	6774
28	TNF	tumor necrosis factor	Extracellular Space	cytokine	7124
29	VIRMA	vir like m6A methyltransferase associated	Nucleus	other	25962
