# Supplementary material for: A novel ND1 mitochondrial DNA mutation is maternally inherited in growth hormone transgenesis in amago salmon (Oncorhynchus masou ishikawae)
Source: Sci Rep. 2022 Apr 25;12:6720. doi: 10.1038/s41598-022-10521-4 (PMC9038734; doi:10.1038/s41598-022-10521-4)
Supplement: Supplementary file 2 — Supplementary Figures. [file 41598_2022_10521_MOESM2_ESM.pdf]

Supplementary figures for Fig. 1(e) Western blotting

A novel *ND1* mtDNA mutation is maternally inherited in Growth Hormone transgenic amago salmon(*Oncorhynchus masou ishikawae*)

Tomohiko Sato, Naoko Goto-Inoue, Masaya Kimisihima, Jike Toyoharu, Ryuhei Minei,
Atsushi Ogura, Hiroyuki Nagoya, Tsukasa Mori

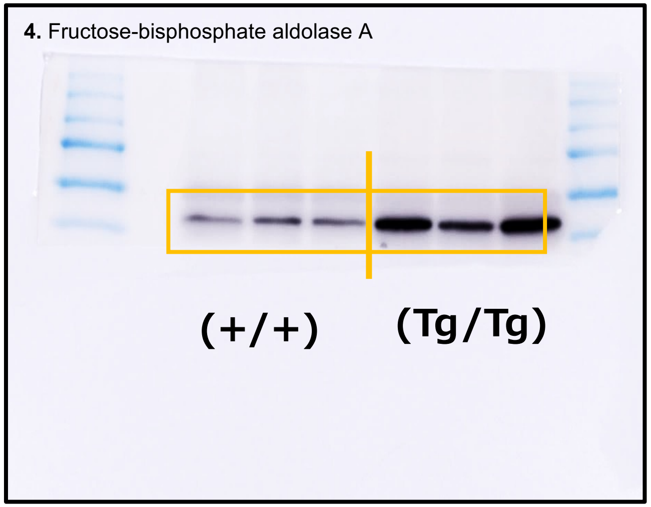

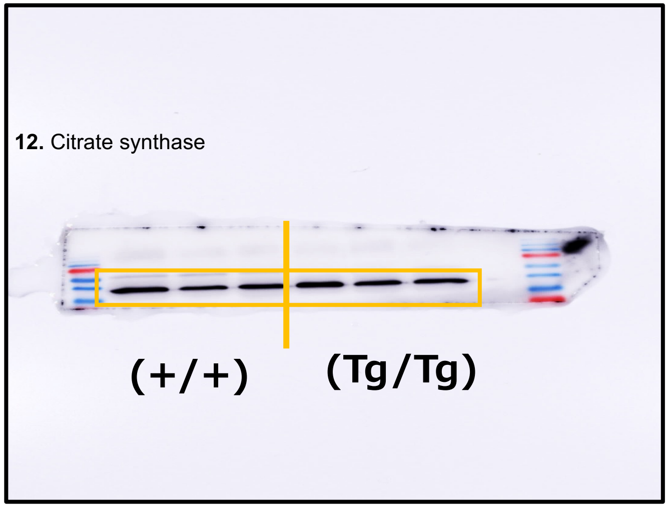

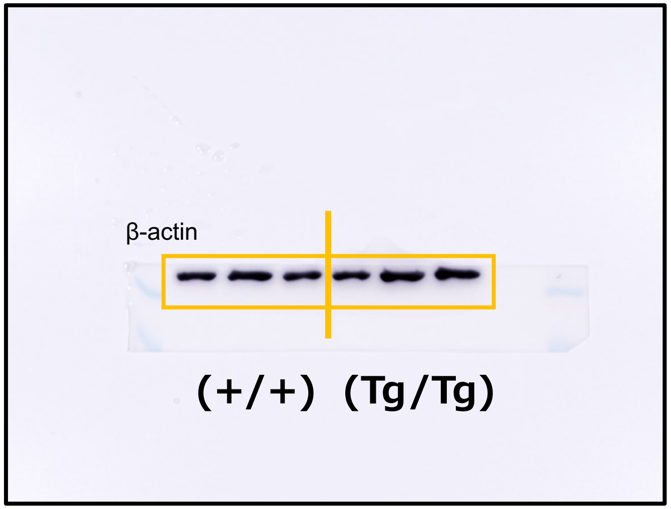

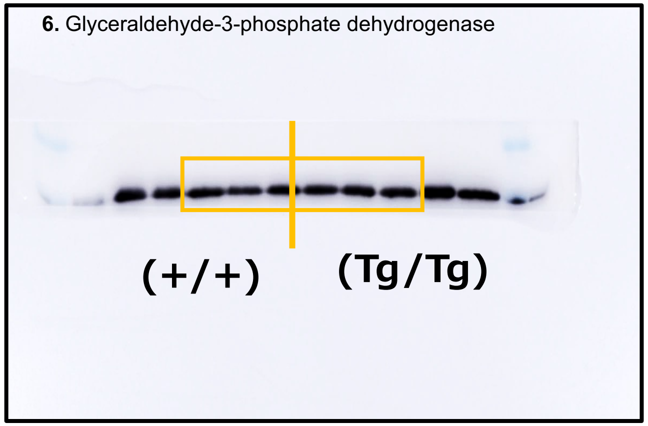

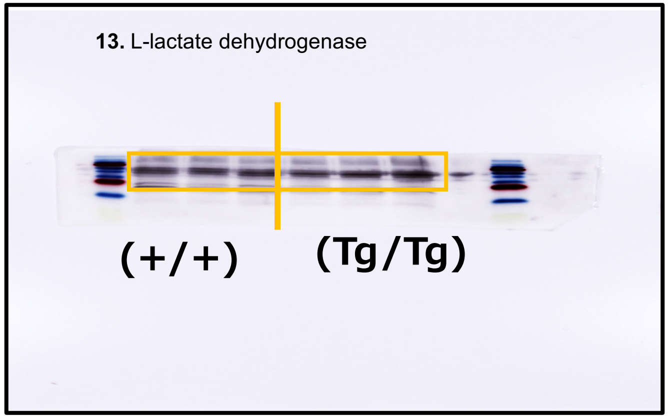

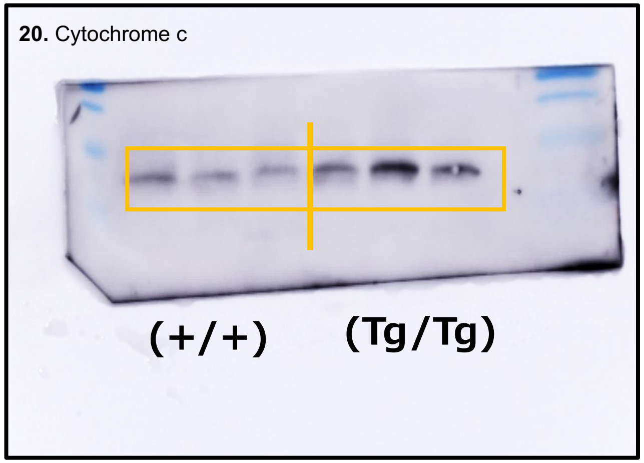
20. Cytochrome c
(+/+)
(Tg/Tg)

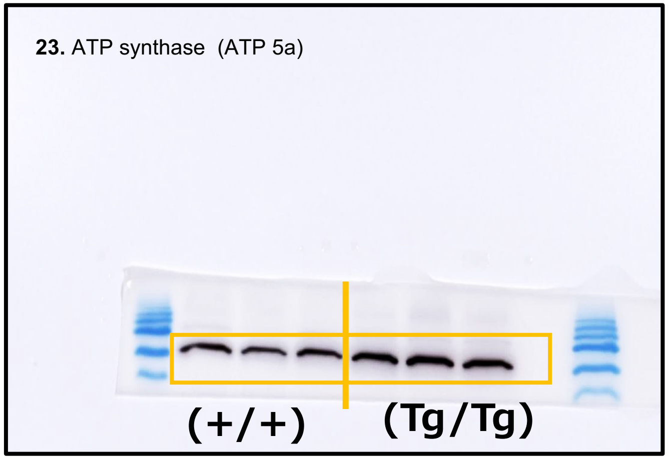
23. ATP synthase (ATP 5a)
(+/+)
(Tg/Tg)

Supplementary figures for Fig. 3(c) OPA1 protein in western blotting

A novel *ND1* mtDNA mutation is maternally inherited in Growth Hormone transgenic amago salmon (*Oncorhynchus masou ishikawae*)

Tomohiko Sato, Naoko Goto-Inoue, Masaya Kimisihima, Jike Toyoharu, Ryuhei Minei, Atsushi Ogura, Hiroyuki Nagoya, Tsukasa Mori

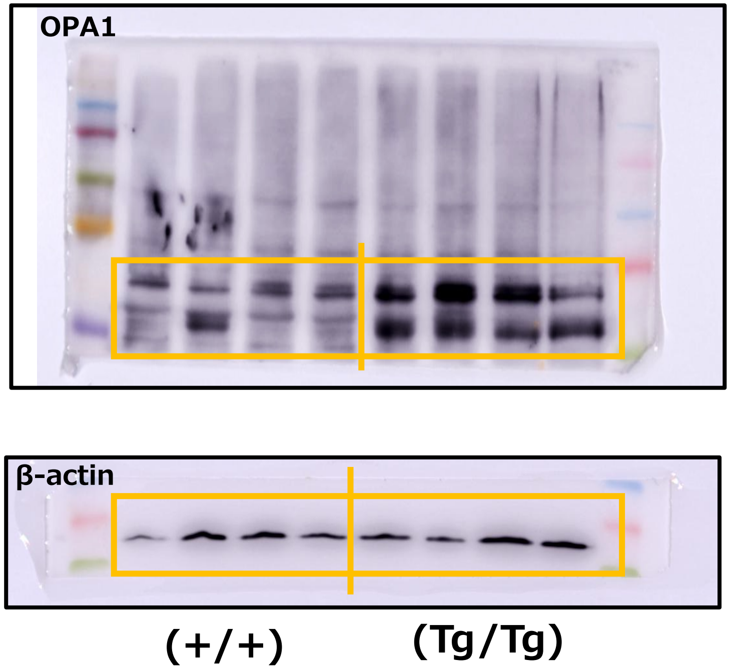

Supplementary figures for Fig. 3(c) PGC-1a protein in western blotting

A novel *ND1* mtDNA mutation is maternally inherited in Growth Hormone transgenic amago salmon (*Oncorhynchus masou ishikawae*)

Tomohiko Sato, Naoko Goto-Inoue, Masaya Kimisihima, Jike Toyoharu, Ryuhei Minei, Atsushi Ogura, Hiroyuki Nagoya, Tsukasa Mori

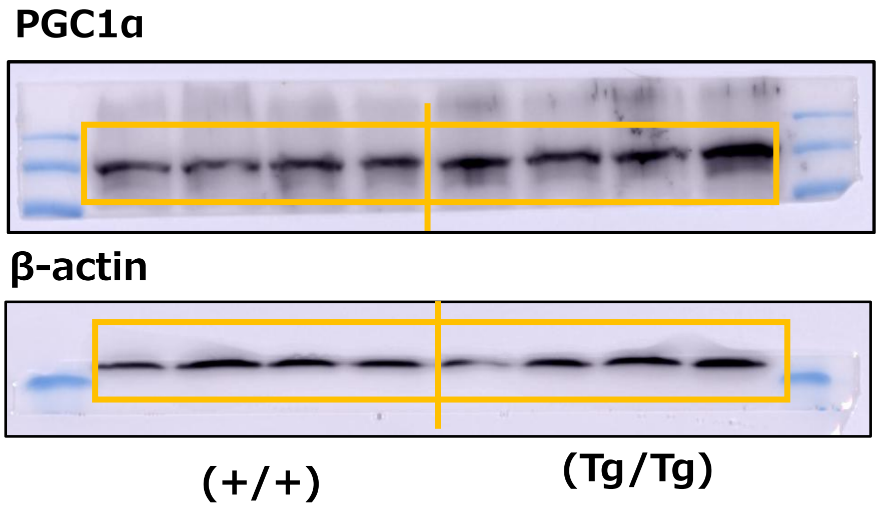

Supplementary figures for Fig. 5(b) western blotting

A novel *ND1* mtDNA mutation is maternally inherited in Growth Hormone transgenic amago salmon (*Oncorhynchus masou ishikawae*)

Tomohiko Sato, Naoko Goto-Inoue, Masaya Kimisihima, Jike Toyoharu, Ryuhei Minei, Atsushi Ogura, Hiroyuki Nagoya, Tsukasa Mori

ND1

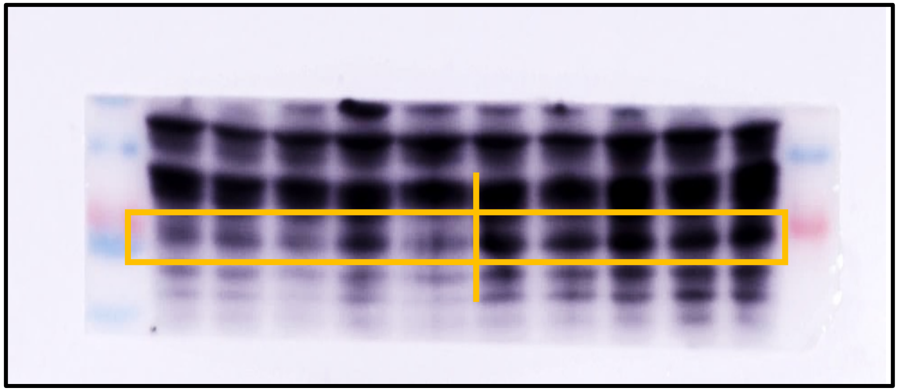

mouseND1

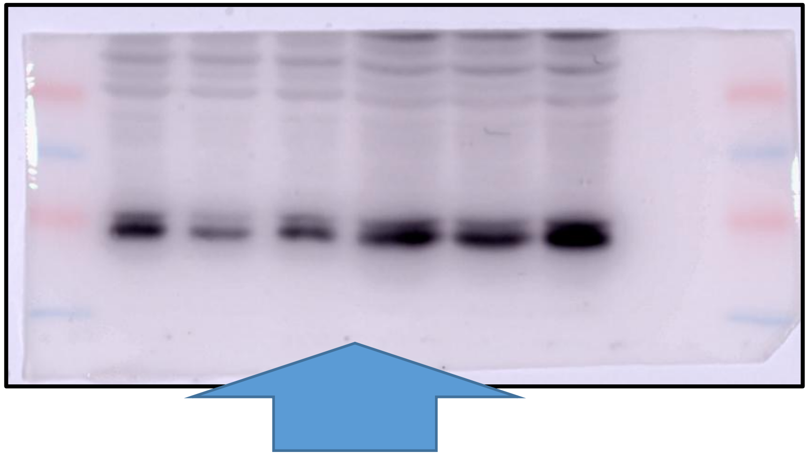

β-actin

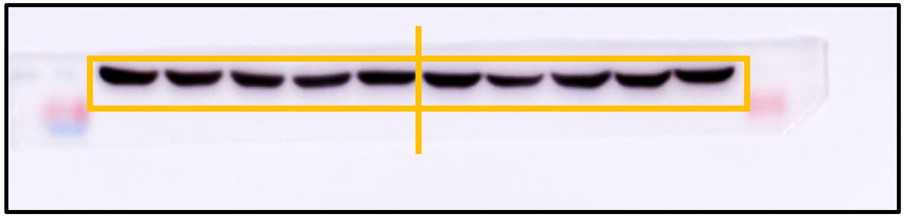

(+/+) **(Tg/Tg)**

In this experiment, we used ND1 antibody for analysis of mouse liver cells.
A strong signal was obtained upon western blotting of the mouse liver proteins; therefore, we used the signal of the amago salmon corresponding to the exact size of the mouse liver.
