# Supplementary material for: A novel ND1 mitochondrial DNA mutation is maternally inherited in growth hormone transgenesis in amago salmon (Oncorhynchus masou ishikawae)
Source: Sci Rep. 2022 Apr 25;12:6720. doi: 10.1038/s41598-022-10521-4 (PMC9038734; doi:10.1038/s41598-022-10521-4)
Supplement: Supplementary file 3 — Supplementary Information. [file 41598_2022_10521_MOESM3_ESM.pdf]

A novel *ND1* mitochondrial DNA mutation is maternally inherited in growth hormone transgenesis in amago salmon (*Oncorhynchus masou ishikawae*)

Tomohiko Sato[1], Naoko Goto-Inoue[1], Masaya Kimisihima[1], Jike Toyoharu[2], Ryuhei Minei[3], Atsushi Ogura[3], Hiroyuki Nagoya[4], Tsukasa Mori[1]*

[1]Department of Marine Science and Resources, Nihon University College of Bioresource Sciences, Japan

[2]Research Institute of Medical Research Support Center Electron Microscope Laboratory. Nihon University, School of Medicine, Japan

[3]Department of Computer Bioscience, Nagahama Institute of Bio-Science and Technology, Nagahama, Japan

[4]National Research Institute of Aquaculture, Fisheries Research and Education Agency, Japan

***Corresponding author:**

Tsukasa Mori

Department of Marine Science and Resources, Nihon University College of Bioresource Sciences, Kameino 1866, Fujisawa 252-0880, Japan

Tel. (81)-466-84-3682; Fax. (81)-466-84-3682

E-mail: mori.tsukasa@nihon-u.ac.jp

Supplementary Information

Supplementary Figures

Western blotting of the mapped enzymes indicated in Fig. 1(e).

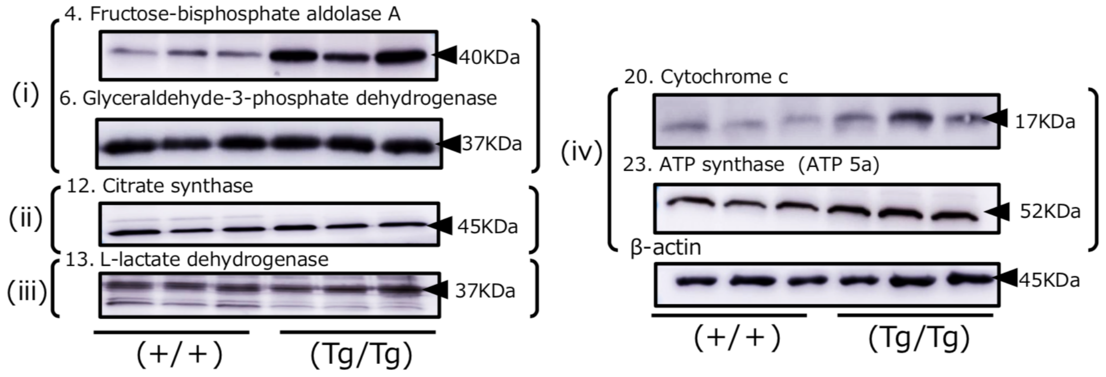

Western blotting of OPA1 and β-actin using four liver tissues from (+/+) and (Tg/Tg) fish.

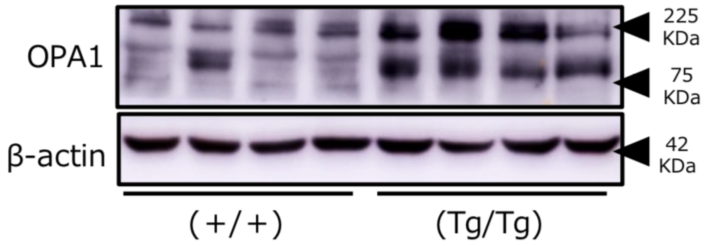

Western blotting of *PGC-1α* and β-actin using four liver tissues from (+/+) and (Tg/Tg) fish.

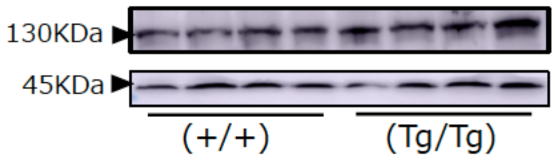

The concentrations of GSSG and GSH in (+/+), (Tg/Tg), (+/Tg), and (Tg/+) fish.

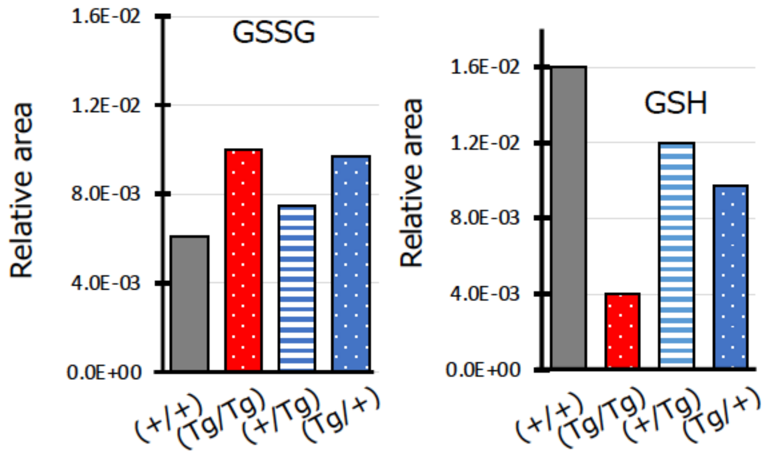

Supplementary Methods

iTRAQ-based proteomic analysis

iTRAQ is a high-coverage proteomic approach used to quantify changes in the abundance of proteins from different sources. Using this method, we aimed to comprehensively analyze the changes in protein abundance in the liver of GH-transgenic amago salmon. Soluble protein was extracted by lysing control and homozygous GH-transgenic amago liver tissues in RIPA buffer (1 M Tris HCl, 5 M NaCl, 10% SDS, 10% Triton X-100; ITSI Bioscience, Johnstown, PA, USA) containing 10 μL/mL protease inhibitor. Proteins were concentrated using acetone precipitation. Protein concentration was measured using the Pierce BCA Protein Assay Kit (Thermo Fisher Scientific, Waltham, MA, USA). Concentration-corrected protein extracts (2.5 μg/μL) were subjected to trypsin digestion (Promega, Madison, WI, USA), followed by

labeling (114:116 = control; 115:117 = Tg/Tg) each protein solution with iTRAQ reagent 4 plex (AB Sciex, Redwood City, CA, USA). The tagged peptide solution was then pooled and concentrated under a SpeedVac. Subsequently, the pooled, concentrated, and dry samples were fractionated using strong cation exchange chromatography to detect trace peptides. Column chromatography samples were re-dissolved in 200 µL of solvent A [50 mM K_2PO_4, 25% acetonitrile (ACN), PH 2.6] and fractionated (Fig. 3, Table 1) using an SCX column (Luna®, 5 µL SCX 100 Å; Phenomenex, Torrance, CA, USA) and HPLC pump (LC-20AD; Shimadzu, Kyoto, Japan). Fractions were then concentrated in a SpeedVac and re-dissolved in sample buffer [20% ACN, 2% trifluoroacetic acid (TFA)]. Thereafter, the re-dissolved samples were desalted using a C18 column (PepClean™ C18 Spin Column # 89870; Thermo Fisher Scientific, Japan). The desalted samples were then eluted with 70% ACN, and the eluate was re-concentrated by evaporation in a SpeedVac. The samples were re-dissolved in 100 µL of solvent B (2% ACN, 0.1% HCOOH) and analyzed via LC with an Easy nano-LC 1000 instrument (Thermo Fisher Scientific). The data obtained by LC-MS/MS were used for protein identification and subjected to quantitative analysis by Thermo Scientific™ Proteome Discover™ 1.4 software (Thermo Fisher Scientific). Detailed procedures and software parameters are described below.

The detailed procedure and software parameters for protein identification and quantitative analysis using LC-MS/MS are as follows:

①Spectrum Selector

(General settings: Use MS1 precursor/Spectrum Properties Filter: Mass range=350–5,000 Da, minimum Peak Count=1/Scan Event Filters: Mass Analyzer=Any, MS Order=Is MS2, Activation Type=Any, Min. Collision Energy=0, MAX. Collision energy=1,000, Scan Type=Is FuLl, Ionization Source=Any, Polarity Mode=Any)

②Sequest HT

(Input Data: Protein Database=Uniprot sprot All 20160125.fasta, Enzyme Name=Trypsin(FuLl), Max. Missed Cleavage Sites=2, Min. Peptide Length=6, Max. Peptide Length=144/Scoring Options: Max. Delta Cn=0.05/Tolerances: Precursor Mass Tolerance=10 ppm, Fragment Mass Tolerance=0.02 Da, Use Average Precursor Mass=False, Use average Fragment Mass=False/Spectrum Matching: Use Neutral Loss (each a, b, y) Ions=True, Weight of each(a, c, x, z)=0, Weight of each(b, y)=1/Dynamic Modifications: Max. Equal Modifications Per Peptide=3, (N,C)-Terminal Modification=None, Dynamic Modification=iTRAQ4plex/+144.102Da(Y), Carboxymethyl/+58.005Da(c)/ Static Modifications: Peptide N-Terminus=

iTRAQ4plex/+144.102Da(Any N-Terminus), Peptide C-Terminus=None, Static Modification= iTRAQ4plex/+144.102Da(K))

③Percolator

(Input Data: Maximum Delta Cn=0.05/ Decoy Database Search: Target FDR (Strict)=0.01, Target FDR (Relaxed)=0.05, Validation based on=q-Value)

④ Reporter Ions Quantifier

(Quantification Method: Quantification Method=iTRAQ4Plex (Custom)/Peak Integration: Integration Tolerance=20 ppm, Integration Method=Most Confident Centroid/Scan Event Filters: Mass Analyzer=FTMS, MS Order=MS2, Activation Type=HCD, Min. Collision Energy=0, Max. Collision Energy=1000)

LC-MS analysis of NAD^+/NADH

Frozen liver tissues (*n* = 3) obtained from four types of fish were weighed (~15 mg) in a 2.0-mL microcentrifuge tube. Liver metabolites were extracted (water:methanol:chloroform = 2:5:2, v/v/v; containing 1 μg/mL HEPES as an internal standard) by single-phase extraction using an ultrasonic homogenizer for 2 min on ice. Further purification steps were performed prior to LC-MS, as described previously[1]. As the NAD^+/NADH data revealed normality and homoscedasticity based on the Shapiro–

Wilk and Levene tests, ANOVA followed by multiple analysis by PLS was performed for statistical analysis.

IPA analysis

Based on the protein identification and quantification data, signal transduction networks in the liver of homozygous GH-transgenic amago were analyzed using IPA software (Ingenuity® Systems, www.ingenuity.com). To investigate the altered metabolic pathways using IPA, salmon protein IDs were re-selected, corresponding to those in a human database (UniProt). The IPA procedure has been described previously in detail[52]. The relationships between phenotype and NADPH oxidase were predicted as previously described[18]. Data analysis was performed by consolidating IDs using the maximum measurement value, with no cut-off for data sets. Content version: 57662101 (Release Date: 2020-09-15).

Primary antibody information

Primary antibodies included anti-fructose-bisphosphate aldolase A (1:1000; #3188; Cell Signaling Technology, Danvers, MA, USA), anti-GAPDH (1:1000; #5174; Cell

Signaling Technology), anti-citrate synthase (D7V8B; 1:1000; #14309; Cell Signaling Technology), anti-L-lactate dehydrogenase B/LDH-B (1:1000; #ab85319; Abcam, Cambridge, UK), anti-cytochrome c (1:500; #MA5-11674; Thermo Fisher Scientific), anti-ATP5a (1:1000; #ab14748; Abcam), anti-OPA1 (1:1000; #bs-11764R Bioss Antibody, Woburn, MA, USA), anti-ND1 (1:000; #sc-293243; Santa Cruz Biotechnology, Dallas, TX, USA), anti-PGC-1α (1:1000; #AB3242; Merck Millipore, Burlington, MA, USA), and anti-β-actin (1:1000; #4967; Cell Signaling Technology).

CE-TOF-MS analysis of liver tissues

Five liver tissues were excised from (+/+) and (Tg/Tg) fish and then immediately frozen in liquid nitrogen. Frozen liver tissue (200 mg) was homogenized in ethanol (500 μL; 4,000 rpm for 60 s, repeated five times)—containing internal standards (Solution ID: 304-1002; Human Metabolome Technologies, Inc., Tsuruoka, Japan)—in liquid nitrogen using a bead homogenizer (Micro Smash MS100R; Tomy Digital Biology Co., Ltd., Tokyo, Japan). After homogenization, 100 μL of each homogenized sample derived from the five fish was pooled (total 500 μL) for further analysis. Thereafter, chloroform (500 μL) and Milli-Q water (200 μL) were added to the sample and mixed well, and the homogenate was centrifuged at 2,300 × *g* and 4 °C for 5 min.

Subsequently, 200 μL of the upper aqueous layer was centrifugally filtered through a Millipore 5-kDa cut-off filter at 9,100 × *g* and 4 °C for 120 min to remove the proteins. Two filters were used per sample. The filtrate was centrifugally concentrated and resuspended in 50 μL of Milli-Q water for CE-MS.

The peaks detected by CE-TOFMS were automatically extracted using the automatic integration software MasterHands ver.2.1.0.1 (developed by Keio University), and the mass-to-charge ratio (m/z), swimming time (MT), and peak area values were obtained as peak information. The peak area values obtained were converted to relative area values using the following formula. Relative area = Peak area of the target sample/[Area value of internal standard × Sample weight (mg)].

Mitochondrial genome analysis

Mitochondria from frozen liver tissues (100 mg) of transgenic and non-transgenic fish were purified using an mtDNA Extractor® CT Kit (FUJIFILM Wako Pure Chemical Corporation, Osaka, Japan). These tissues were homogenized on ice with 1 mL of homogenization buffer using a hand homogenizer. mtDNA was extracted and purified according to the manufacturer’s instructions. One half of the mtDNA was amplified using polymerase chain reaction (PCR) (forward: 5′ -AAC CAA GAC ATT AGA

TTG TGA TTC-3′ and reverse: 5′-TTG GAT TTG CAC CAA GAG TTT TTG-3′). The remaining half of the mtDNA was amplified using another set of primers (forward: 5′-TGA GCC ATA CCT ATT CCT TAC-3′ and reverse: 5′-TTC TTG GGC TAC GTT GAA GGT TTG-3′. PCR was performed using KOD FX Neo (TOYOBO, Osaka, Japan). The reaction mixture was prepared as per the manual, and the following PCR cycling parameters were used: 94 °C for 2 min; 35 cycles of 94 °C for 10 s, 60 °C for 30 s, and 68 °C for 10 min in a T-100 thermal cycler (Bio-Rad Laboratories, Hercules, CA, USA). After PCR, the amplified mtDNA was visualized on an 0.8% agarose (TAE) gel. The concentration of the amplified mtDNA (each half of mtDNA) was measured, and each mtDNA extract (5 μg) was pooled.

Mitochondrial genome assembly

For mitochondrial genome assembly, we obtained short-read sequences from amplified mtDNA extracted from two fish in each of the (+/+) and (Tg/Tg) groups using an Illumina HiSeq X Ten sequencer (Illumina, San Diego, CA, USA). We extracted high-quality reads from the *de novo* assembly pipeline and trimmed low-quality regions from raw fast data using Trimmomatic. Next, high-quality read sequences were assembled *de novo* using the Platanus assembler with default parameters.

Mitochondrial variant analysis using mtDNA

We sequenced mitochondrial genomes from (+/+), (Tg/Tg), (Tg/+), and (+/Tg) fish (three per group) using the Illumina HiSeq sequencer with 150-bp paired-end runs. The sequence reads were trimmed using Trimmomatic. The trimmed reads were mapped using Burrows-Wheeler Aligner (BWA) on the reference mitochondrial genome sequence of *O. masou ishikawae* (NC_008746). After sorting using Samtools, the mitochondrial regions were extracted. Duplicated reads were removed using Picard. Base recalibration was performed using GATK for identifying SNPs and indels. Individual variant calling was performed using GATK HaplotypeCaller. Using this pipeline, we obtained SNP and indel data. We utilized IGV for genomics data visualization and exploration.

Validation of SNPs using RNA-seq

To validate *ND1* variants, one fish from the (Tg/Tg) group was used for RNA-seq. Haplotypes determine the expression of *ND1*, which cannot be cleared using DNA. We sequenced mRNA from the fish samples and processed the sequencing data with AfterQC. We then mapped these reads against the mitochondrial genome (NC_008746) by HiSAT2. *ND1* variants were analyzed using the multiple alignment tool, ClustalW.

Deletion mutation analysis of mtDNA

The deletion mutation sites in (Tg/Tg) mtDNA were investigated using the mitochondrial DNA breakpoints database (Mito Break; mitobreak.portugene.com). To determine the deletion mutation site, BLAST was performed using the *ND1* site of *O. masou ishikawae* and the human homolog. We searched the amino acid residues of the mutated site in amago salmon that correspond to human *ND1* mutations.

Statistical data of concentration of ROS in (+/+), (Tg/Tg), (+/Tg), and (Tg/+) fish determined by d-ROM analysis.

descriptive statistics value					95% Confidence interval for			
Group	N	Mean	Std.Deviation	Std.Error	Lower Bound	Upper Bound	Minimum	Maximum
(+/+)	10	155	32.656	10.327	131.64	178.36	109	231
(Tg/Tg)	10	90.8	30.958	9.79	68.65	112.95	58	158
(+/Tg)	10	116.6	47.552	15.037	82.58	150.62	52	193
(Tg/+)	10	72.2	39.225	12.404	44.14	100.26	41	137

Test of homogeneity of variance			
Leven	df1	df2	P-value
1.799	3	36	0.165

Analysis of Variance					
	Sum Squares	df	Mean square	F value	P-value
Between groups	38587.5	3	12862.5	8.833	0
Within Groups	52421.6	36	1456.156		
Total	91009.1	39			

Multiple comparison		Mean	Std.Error	P-value	95% confidence interval	
(I)Course	(J)Course	Diffrencece(I-J)			Lower Bound	Upper Bound
(+/+)	(Tg/Tg)	64.200*	17.065	0.004	16.55	111.85
	(+/Tg)	38.400	17.065	0.184	-9.25	86.05
	(Tg/+)	82.800*	17.065	0.000	35.15	130.45
(Tg/Tg)	(+/+)	-64.200*	17.065	0.004	-111.85	-16.55
	(+/Tg)	-25.800	17.065	0.836	-73.45	21.85
	(Tg/+)	18.600	17.065	1.000	-29.05	66.25
(+/Tg)	(+/+)	-38.400	17.065	0.184	-86.05	9.25
	(Tg/Tg)	25.800	17.065	0.836	-21.85	73.45
	(Tg/+)	44.400	17.065	0.08	-3.25	92.05
(Tg/+)	(+/+)	-82.800*	17.065	0.000	-130.45	-35.15
	(Tg/Tg)	-18.600	17.065	1.000	-66.25	29.05
	(+/Tg)	-44.400	17.065	0.080	-92.05	3.25

Supplementary References

1. Goto-Inoue, N. *et al.* Mass spectrometry imaging reveals differential localization of natural sunscreens in the mantle of the giant clam Tridacna crocea. *Sci Rep* **10**, 656 (2020).
